# Diurnal variation of the human adipose transcriptome and the link to metabolic disease

**DOI:** 10.1186/1755-8794-2-7

**Published:** 2009-02-09

**Authors:** Andrey Loboda, Walter K Kraft, Bernard Fine, Jeffrey Joseph, Michael Nebozhyn, Chunsheng Zhang, Yudong He, Xia Yang, Christopher Wright, Mark Morris, Ira Chalikonda, Mark Ferguson, Valur Emilsson, Amy Leonardson, John Lamb, Hongyue Dai, Eric Schadt, Howard E Greenberg, Pek Yee Lum

**Affiliations:** 1 Rosetta Inpharmatics, LLC (A wholly-owned subsidiary of Merck & Co., Inc.), 401 Terry Ave N., Seattle, WA 98109, USA; 2 Thomas Jefferson University, Department of Pharmacology and Experimental Therapeutics, Philadelphia, PA 19107, USA; 3 Thomas Jefferson University, Department of Anesthesiology, Philadelphia, PA 19107, USA

## Abstract

**Background:**

Circadian (diurnal) rhythm is an integral part of the physiology of the body; specifically, sleep, feeding behavior and metabolism are tightly linked to the light-dark cycle dictated by earth's rotation.

**Methods:**

The present study examines the effect of diurnal rhythm on gene expression in the subcutaneous adipose tissue of overweight to mildly obese, healthy individuals. In this well-controlled clinical study, adipose biopsies were taken in the morning, afternoon and evening from individuals in three study arms: treatment with the weight loss drug sibutramine/fasted, placebo/fed and placebo/fasted.

**Results:**

The results indicated that diurnal rhythm was the most significant driver of gene expression variation in the human adipose tissue, with at least 25% of the genes having had significant changes in their expression levels during the course of the day. The mRNA expression levels of core clock genes at a specific time of day were consistent across multiple subjects on different days in all three arms, indicating robust diurnal regulation irrespective of potential confounding factors. The genes essential for energy metabolism and tissue physiology were part of the diurnal signature. We hypothesize that the diurnal transition of the expression of energy metabolism genes reflects the shift in the adipose tissue from an energy-expending state in the morning to an energy-storing state in the evening. Consistent with this hypothesis, the diurnal transition was delayed by fasting and treatment with sibutramine. Finally, an *in silico *comparison of the diurnal signature with data from the publicly-available Connectivity Map demonstrated a significant association with transcripts that were repressed by mTOR inhibitors, suggesting a possible link between mTOR signaling, diurnal gene expression and metabolic regulation.

**Conclusion:**

Diurnal rhythm plays an important role in the physiology and regulation of energy metabolism in the adipose tissue and should be considered in the selection of novel targets for the treatment of obesity and other metabolic disorders.

## Background

Circadian (diurnal) rhythms are part of the daily lives of many living organisms, from photosynthetic prokaryotes to higher eukaryotes [1,2]. These oscillations likely evolved to ensure temporal coordination of physiological and behavioral processes, both for adapting to predictable daily environmental changes and orchestrating cellular machinery necessary for life. For example, in cyanobacteria and *Arabidopsis*, the circadian oscillator directs transcription of the photosynthetic machinery to the daylight hours, thereby ensuring the efficient assimilation of light energy [3].

Although first described in the suprachiasmatic nucleus, circadian clocks have been identified in many peripheral tissues, including adipose, heart, kidney and vasculature [4-6]. These peripheral clocks are regulated by central circadian clock machinery and circulating serum markers of circadian function [7,8]. In animal models, many genes in peripheral tissues show oscillatory behavior that is responsive to restricted feeding or other perturbations [9].

The molecular mechanism of the circadian oscillator as a transcriptional-translational feedback loop has been unraveled by genetic analyses in *Drosophila *and mammals [1]. Two transcriptional activators, CLOCK and MOP3/BMAL1, and their target genes, including PER1, PER2, PER3, CRY1, and CRY2, generate a circadian oscillation in their own transcription. Although the core pacemaker involves about a dozen genes, the number of genes that exhibit oscillatory behavior (the circadian output genes) may be much greater. For instance, more than half of the yeast genome is expressed periodically during metabolic cycles [2]. Circadian regulation of genes responsible for basic energy metabolism has also been reported in mice [4-6,10,11].

Alterations of circadian rhythms have been associated with several disease states [8,12,13]. Several epidemiological studies have demonstrated an increased incidence of metabolic syndrome among night shift workers who have chronically disrupted circadian rhythms [14,15]. Supporting evidence comes from CLOCK mutant mice, shown to be hyperphagic and obese and to develop metabolic syndrome in addition to having a disrupted circadian rhythm [16].

Many studies in animals and model systems on the effect of circadian rhythm on gene transcription have been conducted; however, diurnal effects on human tissues are poorly characterized, likely owing to the difficulty associated with non-invasively collecting human tissue samples multiple times/day. Rodent models, while useful, have limitations due to their nocturnal habits and, therefore, certain aspects of the circadian regulation would likely be different from humans. The purpose of this controlled clinical study was to examine the effect of diurnal rhythm on gene expression in the subcutaneous adipose tissue of overweight to mildly obese, healthy individuals and the potential effect of fasting and the anti-obesity drug, sibutramine. We show that remarkably, the expression levels of the core clock genes and the diurnal output genes showed little day-to-day variation during the duration of the study, despite the adipose biopsies being taken from multiple subjects in a trial that lasted over a period of time. Rather, the time of day was the key driver of the expression levels of both core clock and diurnal output genes. We see that diurnal signature was large and consisted of genes involved in growth factor signaling, inflammation and ribosome processing and biogenesis. We also report that both the core clock genes and diurnal output genes were affected by fasting and sibutramine albeit subtly. A connection between growth factors and their inhibitors and adipose metabolism was also observed, leading to the possibility of re-positioning compounds developed for other indications to treat obesity or other metabolic diseases.

## Methods

### Study population and design

Participants were overweight to moderately obese (BMI = 27 to 35 kg/m^2^), healthy, male volunteers, ages 21 to 45 years. Subjects with medical illness, smoking history (6 months prior to study start), history of keloids or bleeding disorder, recent change in weight (+/- 4 kg), or use of prescription medications or NSAIDS two weeks prior to study start were excluded from the study.

**Figure 1:**
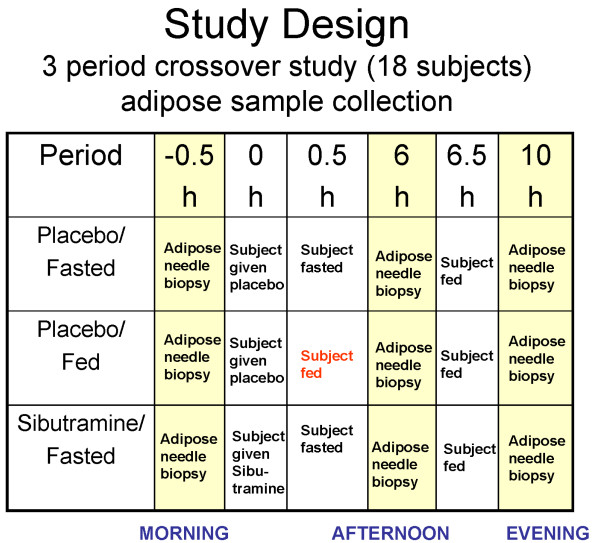
**Schematic of study design**. The first biopsy procedure was performed at 6:30 am to 8:30 am, followed by two more biopsies: one in the afternoon (12:30 pm to 2:30 pm) and one in the evening (4:30 pm to 6:30 pm). The same subject in this crossover design received 9 biopsies over the study duration, with at least 7 days in between visits. In total, 153 adipose samples were obtained and hybridized (17 subjects × 3 time points × 3 periods).

This was a single-site, 3-period, randomized, placebo-controlled, cross-over study (Figure 1). The night before planned adipose biopsies, subjects were admitted to the Phase 1 unit, where standardized meals identical in content and quantity were provided. After the standardized dinner and snack, subjects had an overnight fast. Between 6:30 am and 8:30 am the following morning, subjects underwent a subcutaneous adipose biopsy procedure followed by administration of a single oral dose of 30 mg (3 × 10 mg capsules) sibutramine or placebo. The placebo/fed arm had breakfast 30 minutes post-dose. Between 12:30 pm and 2:30 pm (6 hours post-dose), all subjects underwent a second subcutaneous adipose biopsy procedure followed by a standardized lunch. Between 4:30 pm and 6:30 pm (10 hours post-dose), all subjects underwent a third subcutaneous adipose biopsy procedure. The same subject in this crossover study received a total of 9 biopsies over the duration of the study, with 7 days between visits. Careful recording of the timing of adipose biopsies, food intake and sibutramine/placebo administration was implemented and each participant had biopsies performed by the same physician.

The study protocol conformed to the ethical guidelines of the Declaration of Helsinki, as reflected in approval by the Institutional Review Board of Thomas Jefferson University.

### Biopsy procedure

A small incision in the umbilical region was made to introduce a large bore aspiration cannula (3 mm × 15 cm, Bryon Medical, Tucson, AZ). The cannula was advanced within the anesthetized quadrant while suction from a syringe was maintained. After 1 mL of adipose was collected, after ~6 passes, the cannula was withdrawn. A different quadrant was used each day for collection. The cannula was introduced at each time point using the same incision site. Adipose samples were flash frozen in liquid nitrogen.

### Globin RNA mitigation

To facilitate the collection of multiple human adipose biopsies from a single subject, a minimally invasive method using a syringe was used. Because the syringe method resulted in variable blood contamination in the adipose tissue, Ambion's (Austin, TX) GLOBINclear™ paramagnetic bead capture technology was used to deplete the total RNA sample of globin mRNA, mitigating globin RNA expression to minimize the potential effect of globin contamination on the microarray hybridization.

### Gene expression profiling

Overall, 153 adipose samples were obtained and hybridized (17 subjects × 3 time points × 3 periods). The adipose collection technique was generally well tolerated. Two subjects discontinued during the conduct of the study; one of these subjects was replaced. In total, 17 subjects completed the trial: 13 black, 5 white and 1 mixed race. Evaluation of the globin mitigation technique in a subset of samples demonstrated that the outcome was not markedly different in processed versus non-processed samples, except in smaller signatures, for which the GLOBINclear™ procedure improved the results (Additional files 1 and 2). Data derived from both processed and non-processed samples were used in this analysis.

Total RNA was isolated from the adipose tissues and converted to fluorescently labeled cRNA that was hybridized to Agilent oligonucleotide microarrays [17,18]. The adipose microarray data from this study was deposited into the GEO database under accession number GSE10545. The human gene expression array pattern used was previously deposited in the GEO database (GPL3991).

### Icelandic replication analysis

An independent study was examined to determine whether a common feeding or fasting gene expression signature existed in human adipose tissue. Repeat (1 week apart) biopsies of abdominal subcutaneous fat were isolated from 20 healthy Icelandic subjects. All participants had been fasted overnight (from 9:00 pm) and were randomly assigned to one of two groups: A) The fasted group (n = 10), in which the subject fasted throughout the morning until noon, at which time subcutaneous adipose tissue was collected, or B) The fasted/fed cross-over group (n = 10), in which the subject participated in both a fasting arm (as described in A) and a feeding arm in which the subject consumed a meal between 9:00 am and 10:00 am and subcutaneous adipose tissue was collected two hours later (Additional file 3). Subcutaneous fat samples (5–10 cm^3^) were removed through a 3 cm incision at the bikini line (always from the same site to avoid site-specific variation) after local anesthesia using 10 mL of lidocaine-adrenalin (1%). The incision was closed using a 4/0 vicryl intracutan suture. The adipose tissue samples were placed into aluminum pouches and flash-frozen in liquid nitrogen. A 3 mL alloquot of TRI-Reagent (Molecular Research Center, Inc., Cincinnati, OH) was added to a 600 ± 30 mg piece of fat, and immediately homogenized using an Omni PCR Tissue Homogenizing Kit (OMNI International, Warrenton, VA) for one minute.

### Data analysis

Gene expression data were analyzed using Rosetta Resolver gene expression analysis software (version 7.0, Rosetta Biosoftware, Seattle, WA) and MATLAB (The MathWorks, Natick, MA), following the methods and algorithms developed at Rosetta Inpharmatics [19,20]. To assess the effect of diurnal variation on gene expression and derive a meaningful estimate of the number of genes affected, accounting for the number of false positives due to multiple testing, additional analyses were performed to control for the false discovery rate (FDR; Additional file 4); i.e., the proportion of likely false positives, as previously described [21,22]. The diurnal effect on gene expression was analyzed with a 3-way ANOVA model (time × treatment × patient) via a Monte-Carlo simulation with 100 random permutations. Based on a p-value of 0.01 for ~5000 genes detected in non-permuted data, the expected FDR as estimated by the q-value was ~5% for the mean number of genes satisfying the alpha significance cut-off of < 0.01 among 100 randomizations and the corresponding 95^th ^percentile. A separate analysis was conducted to ensure the adequacy of 100 permutations to provide stable estimates relative to the number of randomizations performed.

A variance filter was applied to remove the genes that showed little to no variation across all experimental conditions to reduce the false discovery rate associated with multiple testing. The filter used was based on the Agilent platform p-value as previously described [20]. 20,000 genes passed the filtering at p < 0.01 and were used for subsequent analyses. The PER1 correlation signature geneset was identified using all the samples in the dataset (Spearman correlation based p-value < 0.01). To compare the gene expression changes related to diurnal rhythm in the different treatment arms, three additional correlations with the PER1 probe were obtained for each of the treatment arms.

Gene function and pathway analysis was performed through the use of Ingenuity Pathways Analysis (Ingenuity^® ^Systems). Canonical pathways analysis identified the pathways that were most relevant to the data set. The significance of the association between the data set and the canonical pathway was measured as a ratio of the number of genes from the data set that map to the pathway divided by the total number of genes that map to the canonical pathway. Additionally, Fisher's Exact test was used to calculate a p-value to determine whether the association between the genes in the dataset and the canonical pathway could be explained by chance alone. The significance of the overlap between gene sets was also determined using Fisher's Exact test under the null hypothesis, stating that the frequency of the signature genes is the same between a reference set of 20,000 genes and the comparison gene sets.

### In silico experiment: correlation between the diurnal signature and the Connectivity Map

To characterize the physiology of diurnal changes in the human adipose, an unbiased *in silico *search for compound signatures common with diurnally regulated genes identified in the present study was performed using the publicly available Connectivity Map database [23]. The Connectivity Map (also known as CMAP) is a collection of genome-wide transcriptional data from cultured human cells treated with different kinds of compounds. The top 200 correlated and 200 anti-correlated probes significantly correlated to the PER1 probe (Spearman correlation based p-value < 10e-15) were selected from the initial PER1 geneset. The probes were then mapped to the U133A probe sets in order to query the Connectivity Map database. In total, 369 U133A probe sets mapped to the selected probes from this study. The connectivity scores and p-values were obtained using CMAP algorithm [23].

## Results

### Diurnally-regulated genes dominate the adipose tissue signature

**Figure 2:**
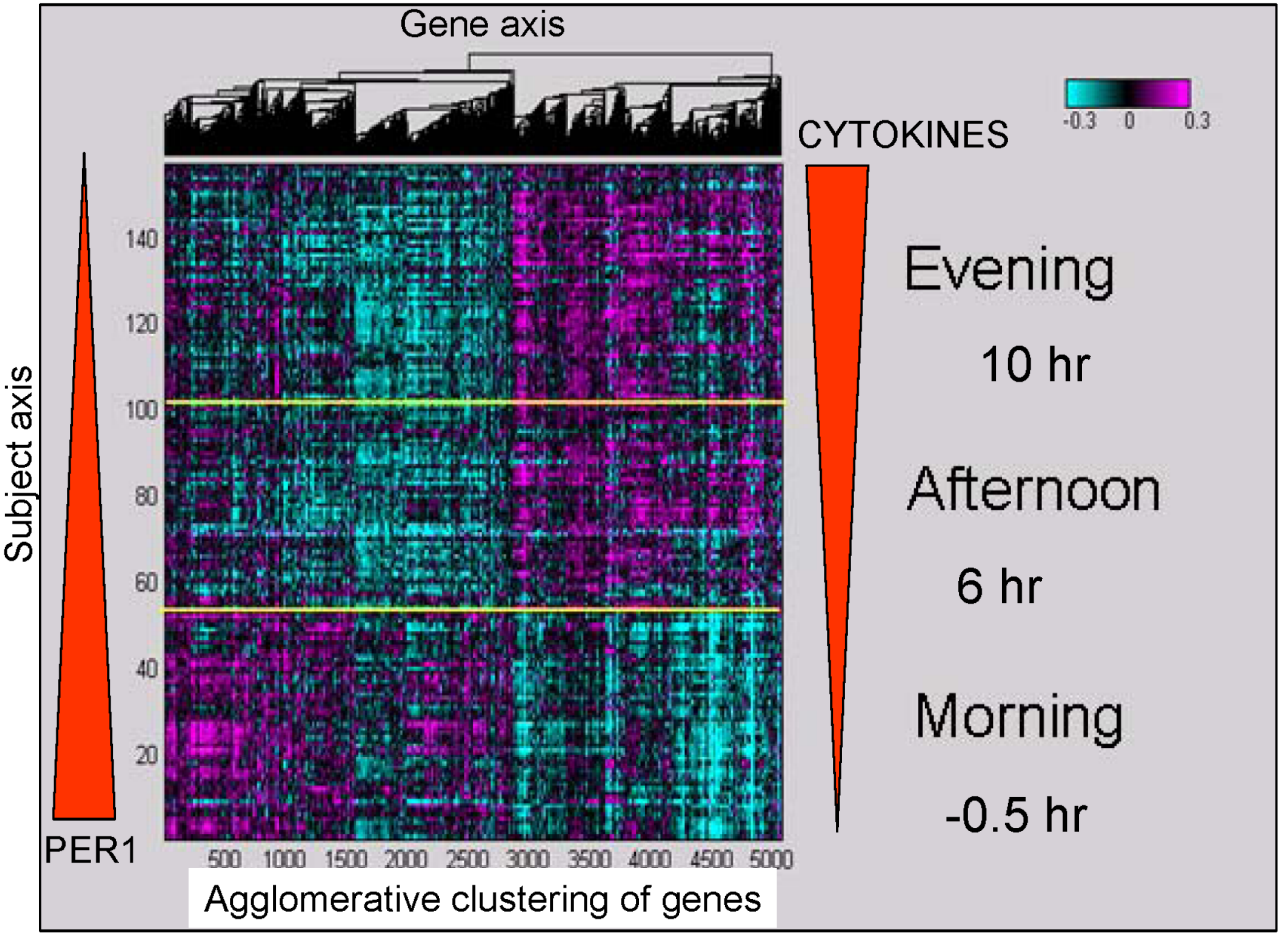
**The impact of diurnal rhythm on global gene expression changes in all subjects**. This heatmap illustrates that more than 5,000 genes were significantly associated with diurnal rhythm in the human adipose tissue over multiple days and subjects, irrespective of treatment arm. PER1 mRNA levels decreased and cytokine mRNA levels increased from the morning to the evening (red block arrows depict levels schematically). The y-axis lists the subjects ordered by time of biopsy and the x-axis lists diurnally regulated genes clustered hierarchically with the agglomerative clustering algorithm. The heatmap color scale is in log_10_, arranged from magenta (upregulated) to cyan (downregulated).

The transcriptional program in the human adipose was largely dominated by the diurnal effect. Figure 1 illustrates a schematic of the study, where biopsies from 17 subjects (BMI 27–35; Age 21–45) were taken in the morning, afternoon and evening. Each subject was admitted to the Phase 1 clinical unit the evening before the start of the trial so that food intake prior to the first biopsy can be properly controlled. The subject then stayed in the clinic throughout the day until all three biopsies were completed. Subcutaneous adipose biopsies were taken around the umbilical region (see Methods for details). This tight clinical setup allowed us to control for variables that might affect gene expression changes not due to the main perturbations. The time of day and patient-to-patient variation had the most profound effect on gene expression, independent of day of biopsy or treatments. To evaluate which factor had the greatest effect on the gene expression observed in the adipose samples, we first performed ANOVA analysis for patient, time and drug treatment on each gene that passed a variance threshold. Among 20,000 genes that passed the variance criteria, 5,194 had p-values < 0.01 for diurnal variation, 6,097 had p-values < 0.01 for inter-patient variation, and 180 had p-values < 0.01 (q-value of ~5%) for treatment (See details in Methods and Additional files 4 and 5). The large but expected inter-patient variability reflects the power of study and the quality of the expression profiles. In addition to the univariate analysis above, we also performed Principal Component Analysis and found the first principal component to be most significantly associated with time of day. Both the univariate and multivariate analyses showed that time of biopsy had a significant effect on gene expression with approximately 5,000 transcripts regulated in a diurnal fashion. Overall, the most profound changes occurred from the morning to the afternoon and the gene expression changes were smaller from the afternoon to the evening (Figure 2).

**Figure 3:**
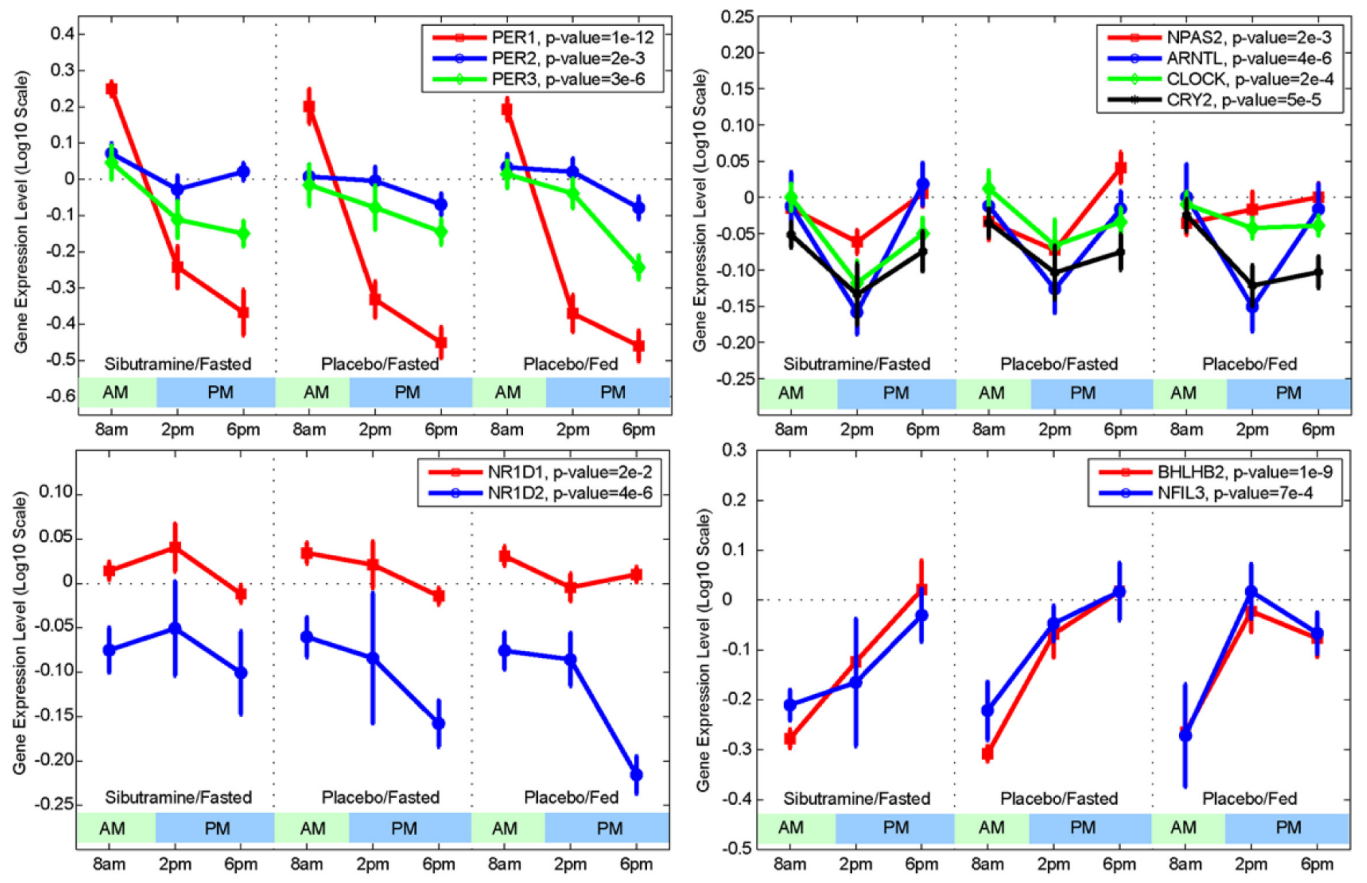
**Temporal profiles of gene expression changes for several core circadian genes **[50]. Each panel illustrates the mean gene expression level (± standard error) across the cohort of patients at 8 am, 2 pm, and 6 pm by treatment arm. The genes are grouped into panels by their temporal profiles and/or similar biological functions. The top left panel shows profiles for PER1, PER2, and PER3 genes. The p-value for time-dependent change is determined by two-way ANOVA for time and patient. Although all three genes show time-dependent changes and consistently have high levels in the morning and low levels in the afternoon across three periods, change in PER1 has the largest amplitude and significance among all core circadian genes. NR1D1 and NR1D2, shown in the bottom left panel, have downward trends, especially from 2 to 6 pm. The genes shown in the top right panel have relatively small temporal changes characterized by a dip at 2 pm, relative to 8 am and 6 pm measurements. The genes in the bottom right panel have a robust upward trend.

The circadian gene, PER1, was prominent among the genes with significantly higher expression in the morning versus the afternoon or evening, with up to a 10-fold change in some patients for PER1 mRNA expression (Figure 3). Known clock genes, including CLOCK, CRY2, BHLHB2 and others, were diurnally regulated in the human adipose (Figure 3). Approximately 5,000 genes were significantly correlated with PER1 mRNA levels (Additional file 6). As expected, significant overlap was observed between the diurnal output gene set from the ANOVA analysis and the PER1 correlated gene set. Genes that were positively correlated with PER1 mRNA levels included those involved in fructose and mannose metabolism and glycolysis (PFKFB, FUK, MPI, PFKM) [4]. Conversely, such genes involved in inflammatory pathways as the cytokines (IL6 and IL8), glucose transporters (GLUT1, GLUT3, GLUT5, GLUT14), cholesterol biosynthesis genes (HMGCR, HMGCS), the low density lipoprotein receptor (LDLR) and genes that control response to free radicals (HMOX1) and hypoxia (HIF1A) were significantly, but negatively, correlated with PER1 mRNA. Transcripts that were most strongly correlated to PER1 were ZNF145 (a zinc finger transcription factor that regulates the renin/prorenin receptor), METRS (involved in rRNA synthesis) and IL6 (a well-known inflammatory gene) [24-26]. Genes previously shown to be diurnally regulated, such as SERPINE1 (PAI-1), were also negatively correlated to PER1 mRNA expression in this dataset [27,28]. Pathways that were enriched in the diurnal signature included inflammatory pathways and the NFKb pathway. Supplemental literature mining processes (Ingenuity) showed that IL-10, IL-6, p38MAPK and PPAR signaling pathways were also enriched (Table 1 and Additional file 7). Tests for biological pathway enrichment in the PER1 correlated genes demonstrated high enrichment for genes involved in RNA processing, ribosome biogenesis and splicing (Table 2).

**Table 1:** Top biological enrichments for the diurnal signature correlated genes

Similar Set	Expectation
response to wounding	3E-19
inflammatory response	9E-16
multi-organism process	3E-14
locomotory behavior	4E-13
taxis	4E-13
chemotaxis	4E-13
response to bacterium	6E-12
response to other organism	6E-12
cytokine-cytokine receptor interaction	2E-10
response to biotic stimulus	4E-10
behavior	2E-09
leukocyte migration	7E-07
blood vessel development	2E-06
vasculature development	2E-06
cell activation	2E-06
leukocyte chemotaxis	3E-06
blood vessel morphogenesis	3E-06

Expectation: p-value after Bonferroni adjustment

**Table 2:** Top biological enrichments for PER1 correlated genes.

Similar Set	Expectation
RNA processing	1E-10
ribosome biogenesis and assembly	4E-06
rRNA processing	6E-06
rRNA metabolic process	9E-06
ribonucleoprotein complex biogenesis and assembly	8E-05
RNA splicing	1E-03
IL1 signaling pathway	2E-03
anti-apoptosis	4E-03
I-kappaB kinase/NF-kappaB cascade	7E-03
response to biotic stimulus	9E-03
negative regulation of apoptosis	1E-02
mRNA metabolic process	1E-02
negative regulation of programmed cell death	1E-02
blood vessel morphogenesis	2E-02
response to virus	2E-02
regulation of cell cycle	2E-02
IL-6 signaling	3E-02
mRNA processing	3E-02

Expectation: p-value after Bonferroni adjustment

**Figure 4:**
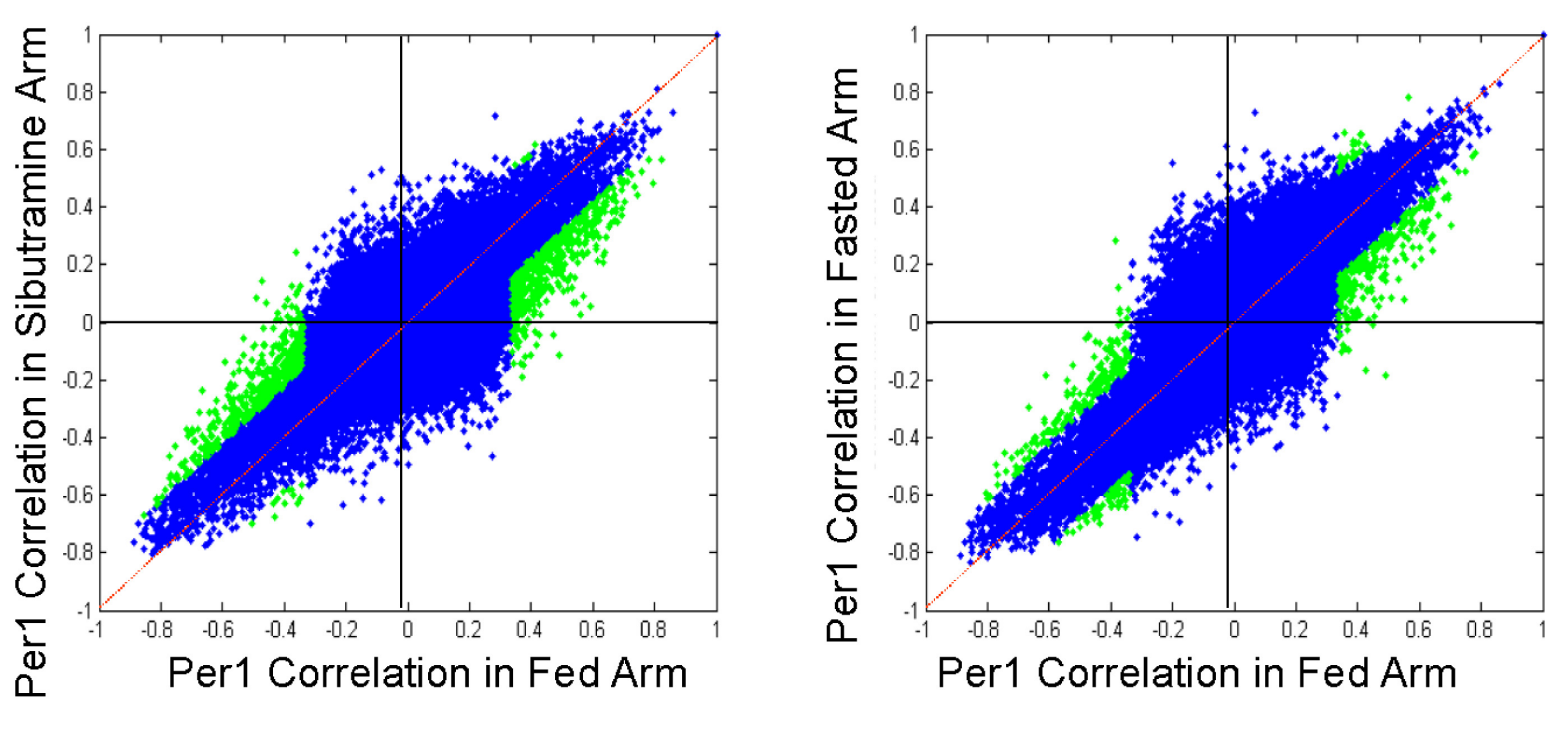
**The majority of the genes in the three states, fed, fasted and sibutramine-treated, were similarly affected by the diurnal signal**. Correlations to PER1 in the three states were very high (blue dots), indicating that the diurnal effect overwhelmed the rest of the perturbations. However, a small number of genes had correlations to PER1 that were significantly different between the states (green dots). The correlation plots are slightly tilted off the diagonal.

Genes regulated in the three states – fed, fasted and sibutramine treated – were similarly affected by the diurnal signal (Figure 4). In the assessment of diurnal regulation by correlation to PER1, a high degree of correlation among the three states was observed, indicating that the largest effect on the transcriptome is the time of day and not any other perturbations; nevertheless, there were ~500 genes with significantly different correlations to PER1 among the three states.

### Impact of food restriction on the transcriptional profile of the adipose tissue

**Figure 5:**
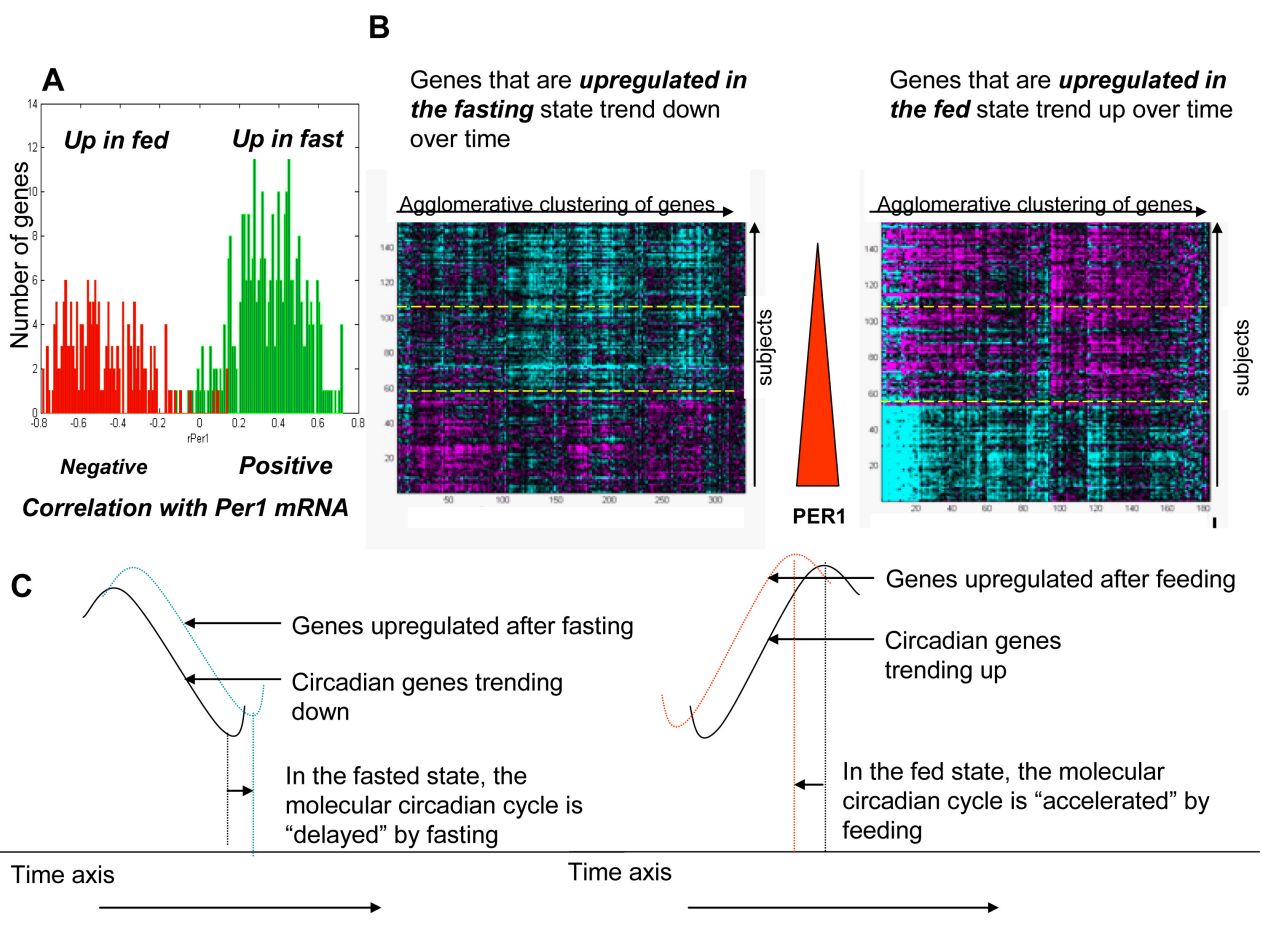
**Genes with expression levels that were changed by fasting and feeding were also diurnally regulated**. A) In general, genes in the fasting signature were positively correlated with PER1 whereas genes in the feeding signature were negatively correlated with PER1. B) These two heatmaps show the trend of these signatures throughout the day. Morning, afternoon and evenings are separated by yellow dotted lines. The color scale of the heatmaps is the same as in Figure 2A. A solid red arrow depicts PER1 mRNA levels declining over time. C) The proposed phase differences produced by fasting and feeding.

**Figure 6:**
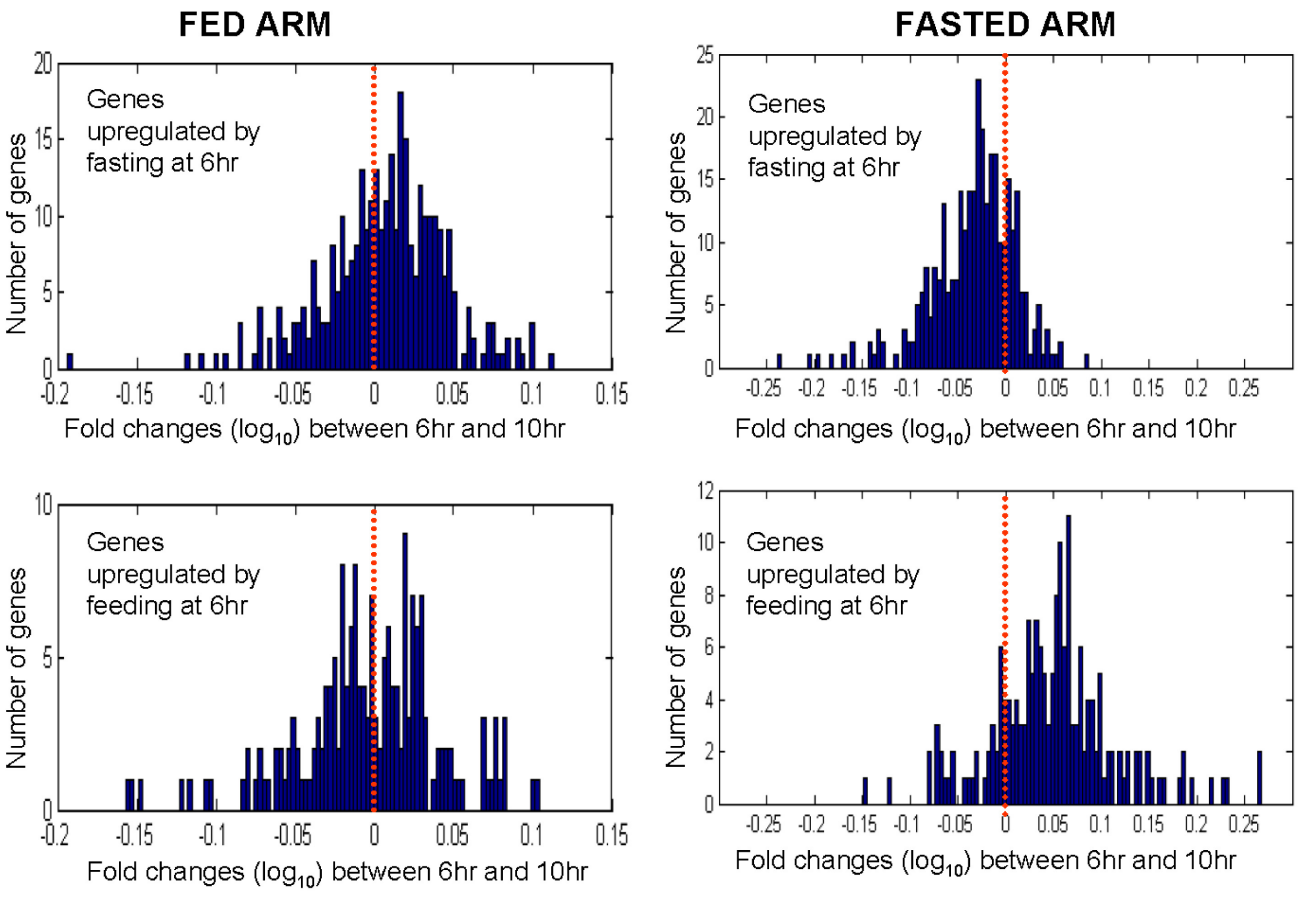
**Genes that were differentially regulated between fasting and feeding at the 6 hour time point continue to be regulated at the 10 hour time point for the fasted arm, but not for the fed arm**. The left panels show that the 6 hour to 10 hour changes for the fast-fed signature in the fed arm mostly center around zero; the right panels show significant changes for the fast-fed signature in the fasted arm. Subjects in all arms were fed 30 minutes after the 6 hour biopsy time point. Y-axis: number of genes. X-axis: Fold changes (log_10_) between 6 hour and 10 hour time points.

Further investigation of genes that were differentially regulated between fasting and feeding at the 6 hour pre-meal time point demonstrated that 498 genes were differentially expressed between the fasted and fed arms (p < 0.01); 318 genes had higher correlations and 180 had lower correlations in the fasted arm compared with the fed arm (Figure 5). Despite the small difference between the fasted and fed arms, the association with the diurnal signature as measured by PER1 correlation was significant (Figure 5). Overall, 94% of the genes with expression levels affected by food intake were correlated to PER1 expression (p < 0.01). In addition, genes that were upregulated in the fasting arm were positively correlated with PER1 expression and genes that were upregulated in the fed arm were negatively correlated with PER1. Because these signature genes were also correlated with PER1, the fasting arm positively affected the genes that were on the diurnal decline (levels trending downward from morning through evening), whereas the fed arm positively affected the genes that were on the diurnal incline (levels trending upward from morning through evening) (Figure 5). Genes that were differentially regulated between the fasted and fed treatment arms at the 6 hour timepoint continued to be regulated at the 10 hour time point in the fasted arm, but not in the fed arm (Figure 6).

Several genes that were regulated by fasting in this study formed a highly connected node in the Ingenuity network, indicating that these genes have been found to be biologically inter-connected in other independent studies. In this tight network, the gene oncostatin (OSM), a macrophage-expressed gene [26], was downregulated > 2-fold in the fasted state compared with the fed state. Forming a network with OSM are other genes that are also downregulated by fasting, such as LDLR, MMP3, EGR1 and IL8. Although the inflammatory genes overall were on the incline with the diurnal rhythm, the inflammatory genes were more downregulated in the fasted state, suggesting that fasting delays the diurnal rhythm by dampening the upward climb of the expression of these inflammatory genes.

### Icelandic replication analysis

With the aim of validating the fasting and feeding signatures of the present study, we analyzed another completely independent study carried out on 20 Icelandic subjects (see Methods and Additional file 3 for details). There was a significant overlap of ~9% of the fasting signatures observed in 20 Icelandic subjects compared with the signatures observed in the current study (p = 1.77 × 10^-11^), despite potential confounding factors such as a slightly different experimental paradigm, ethnicity and population bias. LDLR, a gene in the aforementioned OSM (Oncostatin) network, was among the genes common between these two datasets.

### Impact of the anti-obesity drug, sibutramine, on the transcriptional profile of the human adipose tissue

**Figure 7:**
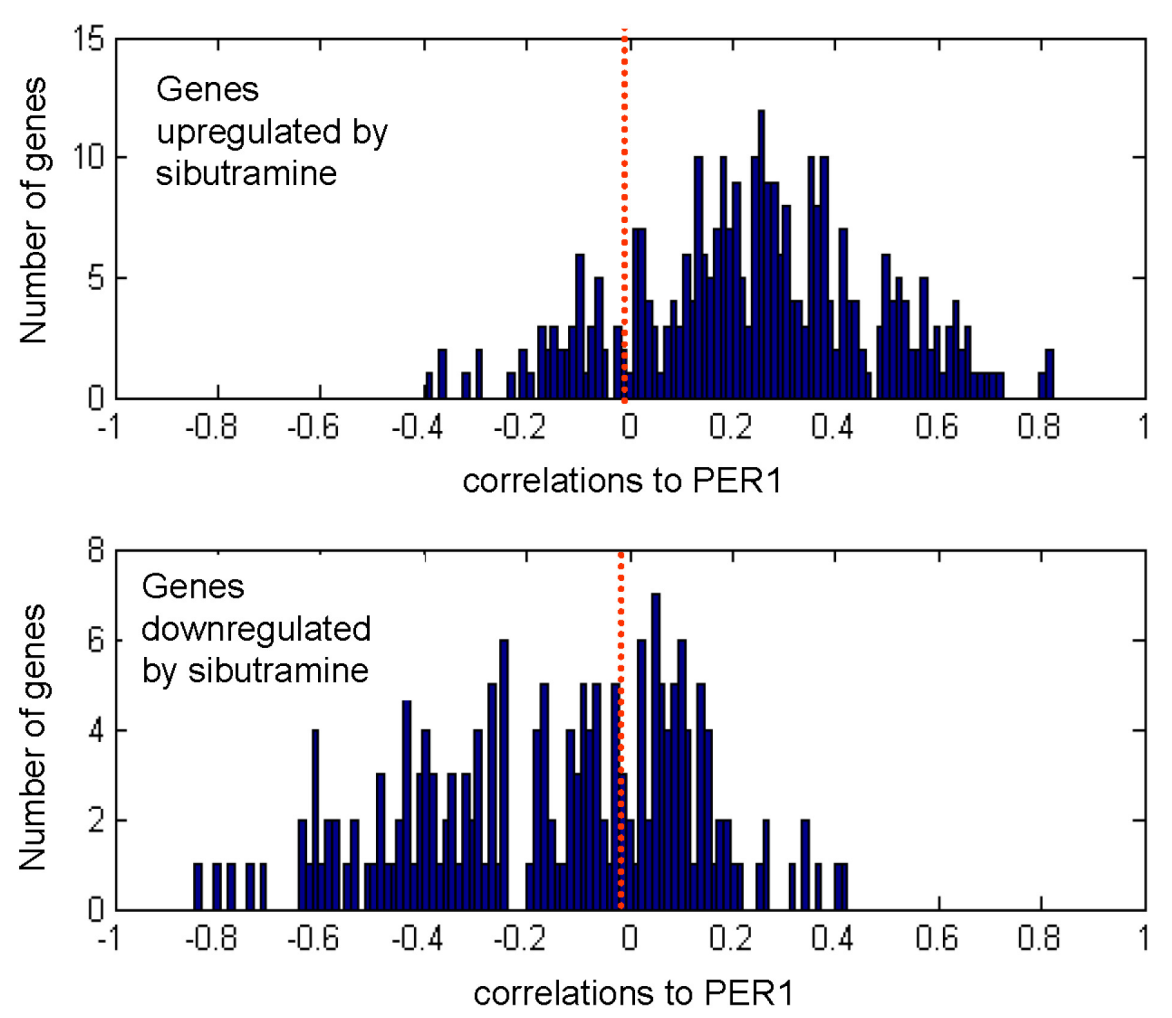
**Differentially regulated genes between the sibutramine treated arm and the fasted arm (both arms started the procedure in a fasted state) at the 10 hour time point**. The top panel shows the correlations to PER1 of genes that are upregulated by sibutramine. The bottom panel shows the correlations to PER1 of genes that are downregulated by sibutramine. The red dotted line marks the zero point. Y-axis: number of genes. X-axis: correlations to PER1.

At 6 hours post-dose (afternoon), 136 genes had differential expression between sibutramine and placebo in the fasted state (p-value < 0.01 by ANOVA), fewer than the 200 genes expected by random chance. At 10 hours post-dose (early evening), 552 genes had differential expression between sibutramine and placebo in the fasted state (p-value < 0.01 by ANOVA). The genes upregulated in the drug treatment arm were positively correlated with PER1 and the downregulated genes were negatively correlated with PER1 (Figure 7), a pattern very similar to what we observed from the effect of fasting signature. There was a differential signature between the placebo arm ("fasted arm") and the drug arm only at the last time point. We note that the subjects would have been fed once 4 hours before the 10 hr time point biopsy (Figure 1). By this time, the fasted arm may have already "caught up" with the fed arm as we do not find any differentially expressed genes between the fasting and fed groups at the 10 hr time point, whereas that difference between those two arms existed at the 6 hr time point. We did, however, find a significant number of differentially regulated genes between sibutramine and placebo at 10 hr, indicating that sibutramine was still actively affecting the diurnal genes and the phase shift is allowing the sibutramine – placebo groups to be different. As shown in Figure 7, these genes are still correlated to PER1 mRNA levels. There was no significance difference between the placebo (fasted) and the sibutramine at the 6 hr time point because they were both equally affecting the diurnal genes in the fasted state.

### Inhibitors of Growth Factor Pathways "reverse" the diurnal signature in silico

In order to investigate what other perturbations will result in similar transcriptional changes to those we observed in the adipose brought on by the diurnal rhythm, we performed an *in silico *experiment, leveraging the publicly available data in the Connectivity Map [23] which contains a collection of signatures elicited by treatment of human cell lines with high doses of many different drugs for 6 hours. The diurnal signature showed the most significant association with the Connectivity Map signatures elicited by drugs that block the PI3K-mTOR pathways such as sirolimus (also known as rapamycin), LY-294002 (selective phosphatidylinositol 3-kinase (PI3K) inhibitor), and wortmanin (also a phosphatidylinositol 3-kinase (PI3K) inhibitor) (Table 3). Sirolimus was also the top hit when the Connectivity Map was queried using the PER1 signature (Additional file 8).

**Table 3:** Drugs with significant enrichment to diurnal signature in the Connectivity Map

rank	drug	mean	n	p-value
1	sirolimus	0.68	10	0
2	LY-294002	0.54	17	0
3	wortmannin	0.62	8	0.0001
4	trichostatin A	0.48	12	0.0012
5	resveratrol	0.61	5	0.0026
6	5182598	-0.78	2	0.0043
7	estradiol	-0.39	10	0.0079
8	iloprost	-0.71	3	0.0105
9	tacrolimus	-0.56	3	0.0153
10	arachidonyltrifluoromethane	-0.75	2	0.02
11	deferoxamine	0.49	3	0.0292
12	fludrocortisone	-0.60	2	0.0429
13	quercetin	-0.53	2	0.0607
14	TTNPB	-0.57	2	0.0645
15	vorinostat	0.53	2	0.0734
16	fulvestrant	0.39	7	0.0858
17	raloxifene	-0.15	3	0.0876
18	rottlerin	0.43	3	0.0945

The drugs with significant connectivity to the diurnal signature are ranked in the order of significance of the connectivity score as judged by the permutation p-value (column labeled "p-value"). Significance cut-off was set at pvalue = 0.1. The permutation p-value estimates the likelihood that the enrichment of a given set of post-dose gene expression changes (instances) by a given drug, relative to all available post-dose changes in the CMAP database (for all drugs) would be observed by random chance. Mean: the arithmetic mean of the connectivity scores for the post-dose changes by the given drug; N: the number of instances of a given drug in CMAP database[23].

## Discussion

The adipose tissue, a major player in energy homeostasis in the body, has a complex mechanism of metabolism regulation, controlled both by internal rhythm and external stimuli, such as food intake. The effect of the circadian rhythm on the transcriptome of the adipose and liver has been described in animal models; however, studies examining diurnal or fasting effects or the effect of anti-obesity drugs on the transcriptome in the human adipose tissue are lacking.

The present study shows that the diurnal effect dominates the transcriptome of the human adipose tissue, with more than 25% of the transcribed genes being diurnally regulated. This finding is consistent with observed circadian regulation in adipose in animal models, in which up to 50% of the genes are under circadian control [5,7,11]. Moreover, the results demonstrated robust regulation of the core clock gene PER1 and of genes encoding for ribosome processing and biogenesis and inflammatory processes. Ribosome biogenesis genes were on the diurnal incline, with levels rising by afternoon and remaining constant until evening. Ribosome biogenesis is an indicator of cellular activity and, in this case, most likely driven by the AKT/PI3K/mTOR pathway [29]. A number of genes encode enzymes in glucose, mannose and fructose metabolism (PFKFB3, FUK, MPI, PFKM), with high expression levels in the morning and a decline in the afternoon through the evening, following the trend of PER1. Conversely, "fuel accumulation" genes, such as those involved in cholesterol biosynthesis (HMGCR, HMGSC1), LDL receptor (LDLR), and glucose transport (GLUTS 1, 3, 5 and 14) have low levels in the morning and rise in the afternoon. Interestingly, there was no observed correlation between the more typical lipogenesis or transporter genes, such as fatty acid synthase (FASN) or GLUTs 2 and 4, and PER1 expression. These genes may be regulated in a more acute fashion by external stimulation, such as insulin or cholesterol, or may not be sensitive to diurnal regulation in the adipose tissue of mildly obese subjects.

Many genes encoding for cytokines and other inflammation-related proteins were also diurnally regulated. The mRNA levels of this set of genes were inversely correlated with PER1 expression, with expression levels increasing dramatically from the morning through the afternoon and being highest in the evening. These genes had among the highest amplitudes of change, from 2-fold (IL-10) to 20-fold (IL-6). Whereas studies have previously shown that inflammation-related proteins, such as PAI-1, IL-6 and TNFα, were diurnally regulated [12,28], the present result adds several new cytokines, including PTX3, IL1, IL10, GRO1, GRO2, CCL6, TGFA and CCL7 to the set of known diurnally regulated genes. Many cytokines, such as IL-6 and IL-8 and MCP-1, have been implicated in cardiovascular risk; the present study demonstrated that both IL-6 and IL-8 were significantly, but inversely, correlated with PER1. The observed associations between these pro-inflammatory genes with the diurnal rhythm warrant further investigation.

To further characterize the physiology of the diurnal change in the human adipose, an unbiased *in silico *search for compound signatures common with the diurnally regulated genes in our study was performed using the publicly available Connectivity Map database. Significant overlap was observed with the AKT/PI3K/mTOR pathway inhibitors, leading to the hypothesis that a signature elicited by insulin or other growth factors would also overlap with the diurnal signature. To test this hypothesis, we used a set of genes that were regulated by treatment of growth factors such as EGF, b-FGF, IGF1, Insulin or Heregulin in MCF7 and HT29 cell lines (Additional file 9; manuscript submitted). As expected, the growth factor pathway genes were correlated with PER1 and the correlations were in the same direction as that of the diurnal set. Moreover, the growth factor gene set linked to the same growth inhibitors from the Connectivity Map query.

The connection between the AKT/PI3K/mTOR pathway and the diurnally regulated adipose tissue is intriguing. Several studies have linked the AKT/PI3K/mTOR pathway to obesity and, independently, the circadian rhythm to metabolic syndrome [16,30,31]. A key kinase in the mTOR pathway is S6K. The S6K -/- mouse is resistant to diet-induced obesity, having adipocytes that do not accumulate lipids [30]. The mTOR pathway is strongly upregulated during adipogenesis [31,32]. The CLOCK mutant mouse has metabolic syndrome [16]. Regulated by AKT and a key player in the AKT/PI3K/mTOR pathway, glycogen synthase kinase 3 beta (GSK-3), the critical checkpoint for glycogen synthesis, is linked to the circadian rhythm [33]. Modulation of GSK-3, also known as *shaggy*, alters circadian rhythms in *Drosophila *and affects clock genes in mammalian cells [34,35]. The findings of the present study are consistent with the connection between the mTOR pathway and the link between circadian rhythm and glucose metabolism. Several cancer drugs that target growth factor pathways might "reverse" the circadian pattern, thus preventing adipose from going into lipid accumulating/anabolic state in the evening. This hypothesis is consistent with the reported side effects of sirolimus, a drug with a significant negative association with circadian rhythm and that leads to hyperlipidemia and accumulation of fatty acids in circulation [36,37], possibly owing to the very high doses necessary, which may prevent the anabolic state of the adipose. Data from various F2 mouse crosses also show that mTOR is causal for obesity traits [38]. Taken together, these observations suggest that anti-cancer drugs, in appropriate doses, may be useful anti-obesity compounds.

Consistent with the observations on the tissue level, the addition of glucose to a rodent cell line led to the down regulation of PER1 and induction of circadian oscillations [39]. In the same model system, oscillations have been induced by the addition of growth factors or prolonged activation of MAPK pathway, and stalled by MEK inhibitors [40]. In addition, BMAL and CLOCK have involvement in glucose homeostasis [41]. These results, together with the findings from the present study, provide support for an association of circadian rhythm with growth factor signaling and metabolic effects.

Another interesting compound uncovered by the *in silico *query of the Connectivity Map was resveratrol, a natural activator of SIRT1 – a circadian deacetylase for core clock components, countering the effect of CLOCK, shown to have histone acetylation activity [42,43]. SIRT1 is also linked to metabolic disease [44]. This result provides additional support for the link between diurnal rhythm and metabolic output. In addition, the diurnal signature clearly overlapped (p = 1.16 × 10^-13^) with a large set of key genes that form an adipose module in a gene-gene correlation network that tested causal for various metabolic endpoints, such as obesity, diabetes and cardiovascular disease [45]. This underscores that genes in the adipose diurnal signature can be mined for drug targets against obesity and other metabolic phenotypes. One of the most correlated PER1 genes in the adipose was ZNF145 (also known as PLZF), which drives metabolic syndrome in rats and affects the transcription of the prorenin/renin receptor [24,46]. A recently implicated gene in humans for obesity, FTO, is also part of the PER1 signature [47].

The physiological changes associated with the diurnal variation of the human adipose transcriptome are important to understand. It is reasonable that humans, like other organisms that live according to the light-dark cycle imposed by earth's rotation, would evolve to compartmentalize energy metabolism in synchrony with diurnal rhythm. We hypothesize that diurnal rhythm in human adipose underlies the transition from a catabolic, energy-releasing state in the morning to an anabolic, energy-storing state in the evening.

A phase shift in circadian rhythm induced by restricted feeding has been reported in animal studies [5]. Similarly, the present study showed that both the fasting state and sibutramine were part of the diurnal signature, indicating that these two perturbations had an effect on the metabolism of the adipose tissue. Both interventions induced a temporal delay in the diurnal rhythm, thereby extending the catabolic state of the adipose (into the afternoon with fasting and into the evening with sibutramine). The delay observed in the fasting arm ended by the evening, likely owing to the fact that subjects were fed in the afternoon. However, the delay caused by sibutramine was evident at the last time point, even after feeding, indicating that sibutramine was still actively affecting the diurnal genes. One of the most differentially down regulated genes in the sibutramine versus the fasted arm at the 10 hour time point was chemokine (C-X-C motif) ligand 1 (CXCL1), a secreted cytokine involved in numerous inflammatory pathways. CXCL1 is also known as growth-regulated oncogene-alpha (GRO-alpha) and is involved in many tumor types as an oncogene [48,49].

Differences between the regulation of clock genes in humans versus rodents have been observed. Contrary to the results in human adipose in this study, expression of PER1 mRNA in rodents increased from the morning through the evening [50]. In addition, the effect of fasting and sibutramine in human adipose tissue was subtle; again, quite different than in rodents, for which restricted feeding produces profound effects on the peripheral clock [51]. However, compared with the present study conducted in overweight to mildly obese humans in the course of one day, many of the rodent studies were conducted in lean mice and the restricted feeding regimen was conducted over many days, potentially confounding the comparison between rodents and humans. Rhythmic expression of clock genes is attenuated in the perigonadal adipose tissues of obese KK mice and obese, diabetic KK-A^y ^mice, indicating that obesity and disease state are intricately linked to the circadian rhythm [6]. To further investigate the differences between rodent models and humans and the association between obesity and circadian rhythm in humans, a similar study in lean and morbidly obese individuals could be conducted. Nevertheless, despite the limitations noted, the major finding is that many genes in the peripheral tissues, such as the adipose in both rodents and humans, exhibit rhythmic expression. The circadian output genes are also linked to metabolism in both species and are affected by such stimuli as restricted feeding. Rodent studies, examining white and brown adipose tissue, liver and skeletal muscle, also showed the number of genes under circadian regulation ranging from 3% to 26%, suggesting that a large proportion of the transcriptome is under circadian control [5,10]. The estimation from the current study is closer to the upper bound of what has been observed in rodents. This could simply reflect the differences in the experimental designs and statistical power to detect changes. On the other hand, it could be attributed to the dominant role of the adipose in driving peripheral clocks in overweight individuals. Clocks in peripheral tissues can be entrained by feeding [52]. One can speculate that feeding patterns in humans may play a substantial role in the synchronization of SCN-controlled and food entrainable oscillations. This synchronization may lead to more efficient energy utilization by adipose and, in turn, may explain the effect of clock-related genes, such as Nocturnin, on resistance to diet-induced obesity [53]. Understanding cross-species similarities and differences is necessary for a deeper understanding of how circadian rhythm affects physiology on the whole.

## Conclusion

To our knowledge, this is the first genome-wide gene expression profiling study of clinical human adipose samples. The results offer new insights into the physiology of adipose tissue in relation to the diurnal cycle, underscoring the importance of diurnal rhythm for basic physiology of the adipose tissue and energy metabolism in the body. It provides a deeper understanding into the connection between diurnal rhythm, energy metabolism, and growth factor signaling. Consistent with previous reports, the present findings suggest that the genes linked to PER1-led oscillations may be exploited as novel points of intervention for obesity and other metabolic phenotypes. A thorough understanding of diurnal effects on energy metabolism and the link to adipose physiology is important for the selection of novel targets for the treatment of obesity.

## Competing interests

AL, MN, CZ, YH, XY, CW, MM, IC, MF, VE, JL, HD, ES and PYL are employees of Merck & Co., Inc. BF is currently an employee of Genentech.

## Authors' contributions

AL and PYL are the main authors for the analysis, interpretation of data and manuscript writing. WKK, JJ, BF, IC, MF, MM and HG conducted or contributed to the clinical study. MN, CZ, XY, YH and HD contributed to the analysis. VE, AL, JL and ES contributed to the Iceland study. All authors read and approved the final manuscript.

## Pre-publication history

The pre-publication history for this paper can be accessed here:



## Supplementary Material

Additional file 1 **Heatmap showing differentially expressed genes between the fasted and fed arms before and after the GlobinClear procedure, used in an attempt to mitigate the hemoglobin contamination from blood. **Hemoglobin contamination has resulted in spurious hybridizations in microarray experiments. Old NGC: first hybridization results without GlobinClear; New NGC: second hybridization results without GlobinClear; New GC: second hybridization results with GlobinClear. Click here for file

Additional file 2 **ROC analysis for derived signature before and after the GobinClear procedure.** Blue solid line: fasting signature, no GlobinClear; Blue dotted line: fasting signature, GlobinClear; Red solid line: sibutramine signature, no GlobinClear; Red dotted line: sibutramine signature, GlobinClear. Some improvement by the procedure was observed in the fasting signature, but not for the sibutramine signature. Click here for file

Additional file 3 **A fasting-feeding study carried out on Icelandic subjects.** (A) Two biopsies from 10 healthy donors separated one week apart for which all subjects had been fasting from 9 pm on day 1 to noon on day 2, at which time the sample collection occurred. (B) Two biopsies from 10 healthy donors entering a two-arm randomized cross-over study, in which subjects participated in both a fasting arm (see A) and a feeding arm, in which subjects consumed a meal between 9 am and 10 am and subcutaneous adipose tissue was collected two hours later. Click here for file

Additional file 4 **Estimation of False Discovery Rate (FDR).** Monte-Carlo simulation for the diurnal signature was carried out by doing 100 random permutations on the gene expression data and performing a 3-way ANOVA. Panel A illustrates the relationship between the number of probes significant at given p-value level vs. the alpha level as observed on the original (non-randomized) data (red line) and the mean of 100 randomizations (blue line). Panel B depicts the estimated q-value vs. the p-value cut-off. Panel C displays the number of significant probes vs. the estimated q-value for a given p-value significance threshold. Panel D shows the relationship between the estimated mean number of false positives vs. the number of significant probes for a given p-value cut-off. Data are shown for the range of p-values up to 0.05 for all 4 panels. Click here for file

Additional file 5 **Transcripts that are significantly differentially expressed with time of biopsy.** ANOVA p-value for time of biopsy less than 0.00001. Click here for file

Additional file 6 **Transcripts that are significantly correlated with PER1 probe.** Absolute value of Spearman correlation coefficient higher than 0.4. Click here for file

Additional file 7 **An enrichment analysis performed in Ingenuity using the diurnal signature and shows the pathways enriched as computed by literature networks.** Click here for file

Additional file 8 **Scatter plot of the Connectivity MAP (CMAP) connectivity score vs. the connectivity score-based rank as provided by the CMAP algorithm.** The gene set correlated with PER1 was queried against ~6000 different treatments, comprised of various treatments and doses in the CMAP database The green curve represents treatments with positive scores (resulting in overall downregulation after treatment); the red curve represents treatments with negative scores (resulting in overall upregulation after treatment), and the gray portion of the curve represents inconsistent or insignificant direction of change post-dose. Sirolimus treatments with various concentrations are indicated by white markers. Click here for file

Additional file 9 **Transcripts that were found in previous studies to be significantly regulated by various growth factors in mammalian cell lines.** Click here for file
