# Supplementary figures and images for: Diurnal variation of the human adipose transcriptome and the link to metabolic disease
Source: BMC Med Genomics. 2009 Feb 9;2:7. doi: 10.1186/1755-8794-2-7 (PMC2647943; doi:10.1186/1755-8794-2-7)

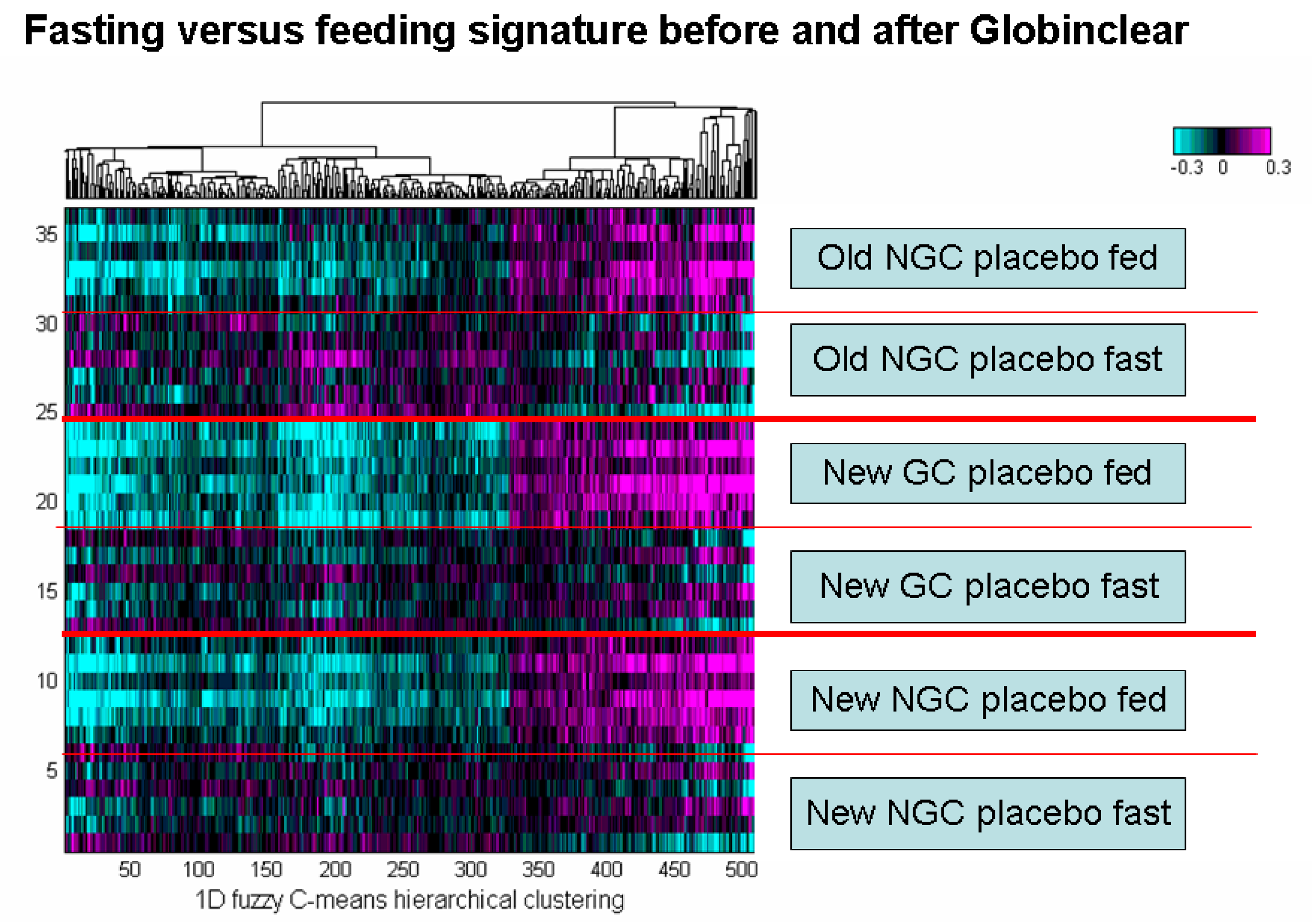

Supplement: Additional file 1 — Heatmap showing differentially expressed genes between the fasted and fed arms before and after the GlobinClear procedure, used in an attempt to mitigate the hemoglobin contamination from blood. Hemoglobin contamination has resulted in spurious hybridizations in microarray experiments. Old NGC: first hybridization results without GlobinClear; New NGC: second hybridization results without GlobinClear; New GC: second hybridization results with GlobinClear. [file 1755-8794-2-7-S1.tiff]

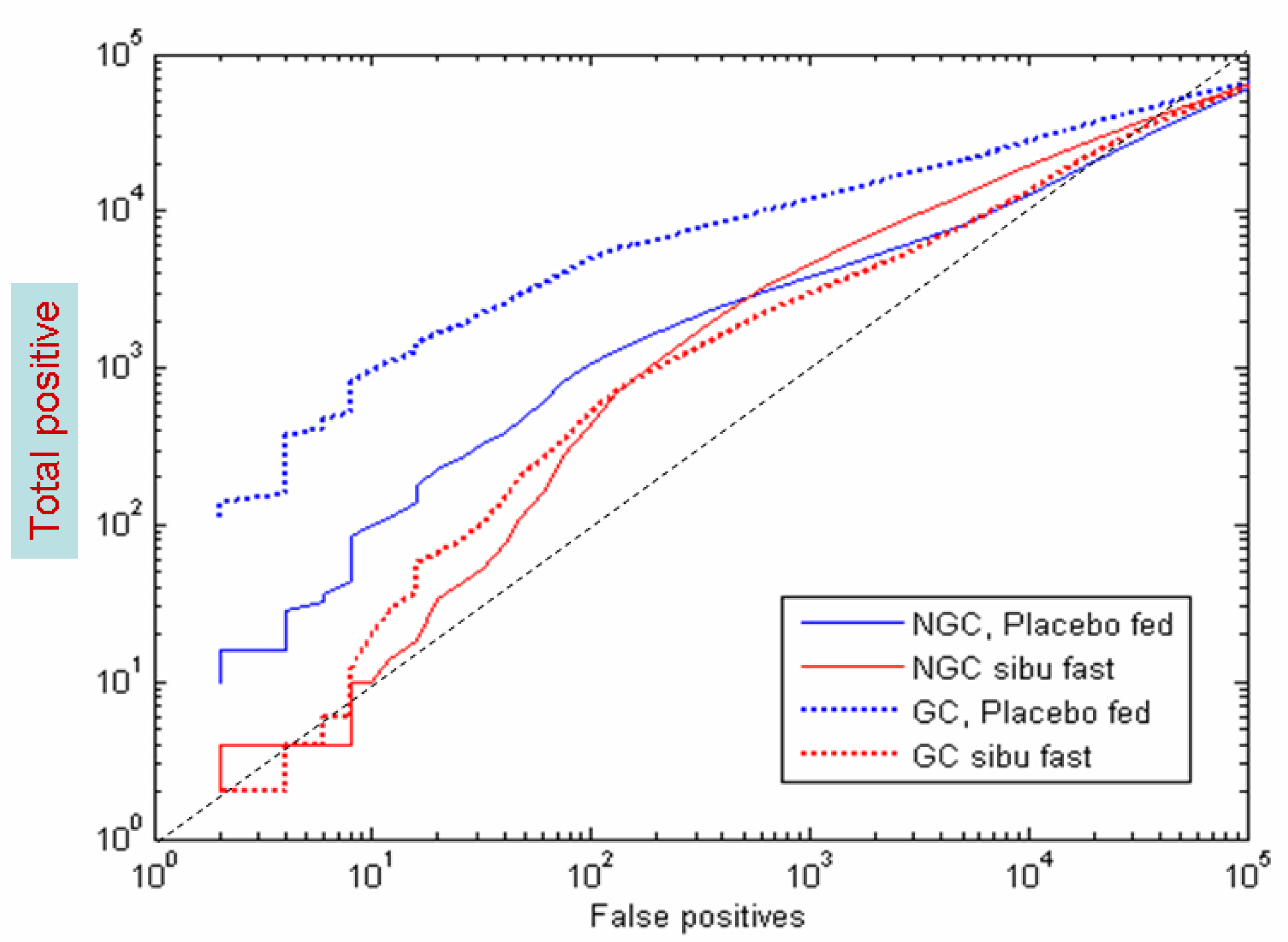

Supplement: Additional file 2 — ROC analysis for derived signature before and after the GobinClear procedure. Blue solid line: fasting signature, no GlobinClear; Blue dotted line: fasting signature, GlobinClear; Red solid line: sibutramine signature, no GlobinClear; Red dotted line: sibutramine signature, GlobinClear. Some improvement by the procedure was observed in the fasting signature, but not for the sibutramine signature. [file 1755-8794-2-7-S2.tiff]

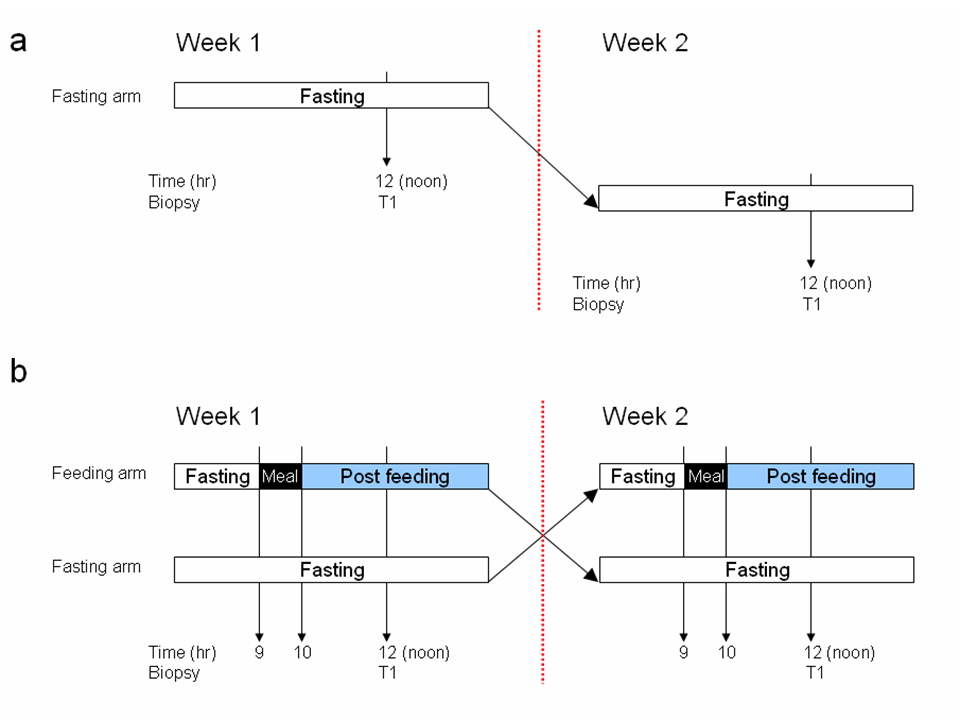

Supplement: Additional file 3 — A fasting-feeding study carried out on Icelandic subjects. (A) Two biopsies from 10 healthy donors separated one week apart for which all subjects had been fasting from 9 pm on day 1 to noon on day 2, at which time the sample collection occurred. (B) Two biopsies from 10 healthy donors entering a two-arm randomized cross-over study, in which subjects participated in both a fasting arm (see A) and a feeding arm, in which subjects consumed a meal between 9 am and 10 am and subcutaneous adipose tissue was collected two hours later. [file 1755-8794-2-7-S3.tiff]

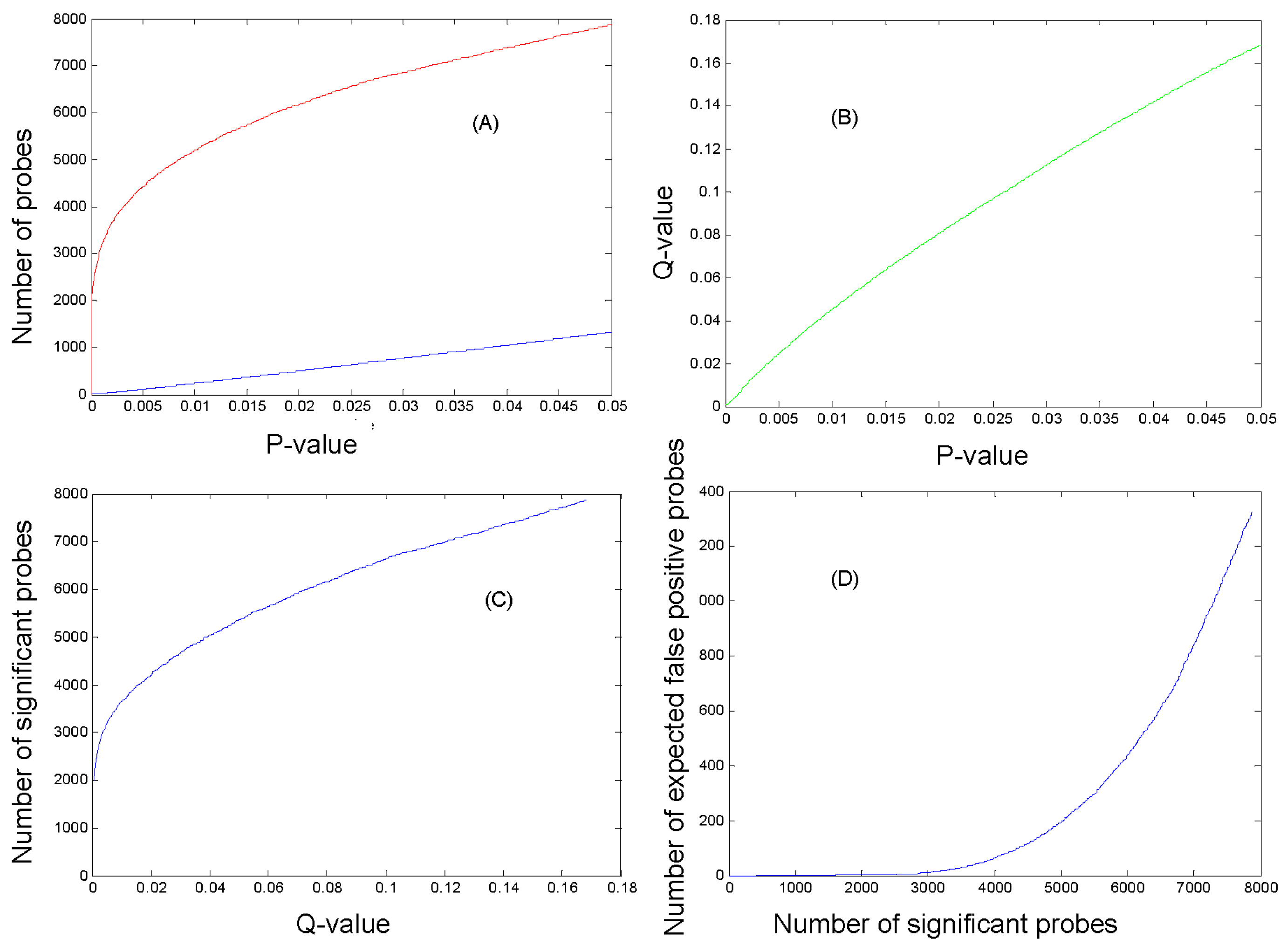

Supplement: Additional file 4 — Estimation of False Discovery Rate (FDR). Monte-Carlo simulation for the diurnal signature was carried out by doing 100 random permutations on the gene expression data and performing a 3-way ANOVA. Panel A illustrates the relationship between the number of probes significant at given p-value level vs. the alpha level as observed on the original (non-randomized) data (red line) and the mean of 100 randomizations (blue line). Panel B depicts the estimated q-value vs. the p-value cut-off. Panel C displays the number of significant probes vs. the estimated q-value for a given p-value significance threshold. Panel D shows the relationship between the estimated mean number of false positives vs. the number of significant probes for a given p-value cut-off. Data are shown for the range of p-values up to 0.05 for all 4 panels. [file 1755-8794-2-7-S4.tiff]

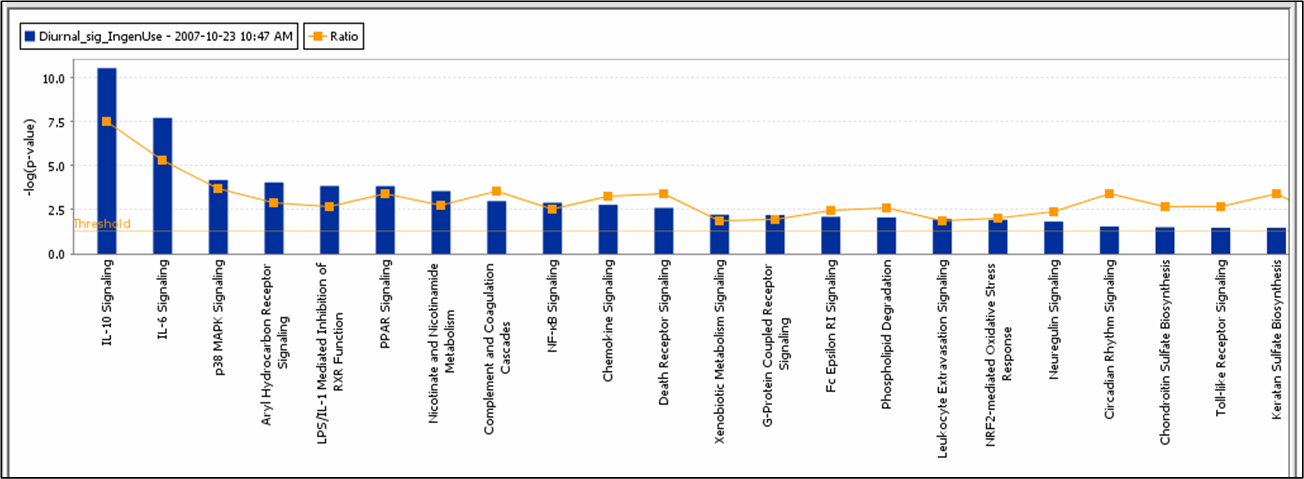

Supplement: Additional file 7 — An enrichment analysis performed in Ingenuity using the diurnal signature and shows the pathways enriched as computed by literature networks. [file 1755-8794-2-7-S7.tiff]

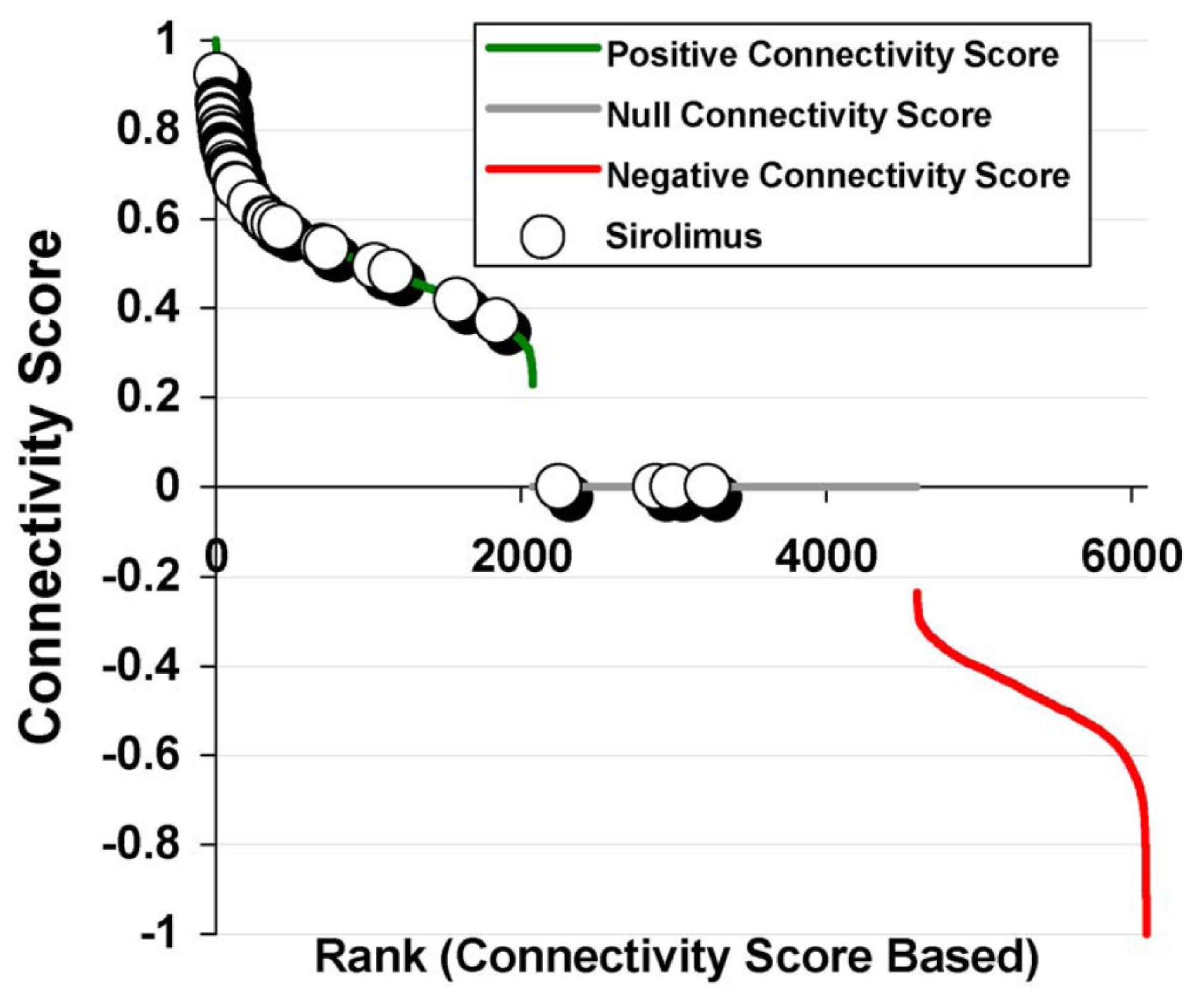

Supplement: Additional file 8 — Scatter plot of the Connectivity MAP (CMAP) connectivity score vs. the connectivity score-based rank as provided by the CMAP algorithm. The gene set correlated with PER1 was queried against ~6000 different treatments, comprised of various treatments and doses in the CMAP database The green curve represents treatments with positive scores (resulting in overall downregulation after treatment); the red curve represents treatments with negative scores (resulting in overall upregulation after treatment), and the gray portion of the curve represents inconsistent or insignificant direction of change post-dose. Sirolimus treatments with various concentrations are indicated by white markers. [file 1755-8794-2-7-S8.tiff]
