# Supplementary material for: Diurnal variation of the human adipose transcriptome and the link to metabolic disease
Source: BMC Med Genomics. 2009 Feb 9;2:7. doi: 10.1186/1755-8794-2-7 (PMC2647943; doi:10.1186/1755-8794-2-7)
Supplement: Additional file 5 — Transcripts that are significantly differentially expressed with time of biopsy. ANOVA p-value for time of biopsy less than 0.00001. [file 1755-8794-2-7-S5.pdf]

| Transcript | pval |
|---|---|
| NM_004920 | 0 |
| NM_080282 | 0 |
| NM_172346 | 0 |
| NM_000392 | 0 |
| NM_016818 | 0 |
| NM_152924 | 1.88156E-10 |
| Contig56303_RC | 1.84514E-11 |
| NM_005158 | 0 |
| NM_138326 | 0.000858865 |
| NM_004458 | 4.49857E-05 |
| NM_022977 | 5.27636E-09 |
| NM_001109 | 0 |
| NM_006988 | 2.32894E-10 |
| NM_020249 | 0 |
| NM_182920 | 4.77396E-14 |
| NM_001124 | 2.88166E-09 |
| AF086149 | 0 |
| AF086543 | 3.10766E-11 |
| NM_024524 | 2.23944E-11 |
| NM_000685 | 2.9754E-14 |
| NM_004835 | 0 |
| AK001060 | 1.19904E-14 |
| AK001808 | 7.16573E-11 |
| AK023363 | 4.46728E-11 |
| AK023526 | 0 |
| AK054990 | 0 |
| AK055302 | 2.24232E-12 |
| AK056146 | 1.25921E-07 |
| AK057578 | 0 |
| NM_000693 | 5.83787E-05 |
| NM_001634 | 0 |
| NM_153206 | 1.90026E-07 |
| NM_139314 | 2.07057E-13 |
| NM_173505 | 3.21965E-15 |
| NM_145699 | 0 |
| NM_030882 | 2.58105E-12 |
| NM_145637 | 1.27923E-11 |
| NM_020979 | 0 |
| NM_020980 | 2.33749E-06 |
| NM_001657 | 0 |
| NM_021069 | 0 |
| AF038193 | 1.08821E-11 |
| NM_178815 | 6.32827E-15 |
| NM_177949 | 2.46569E-12 |
| NM_001674 | 1.11022E-16 |
| NM_004024 | 0 |
| NM_033027 | 0 |
| NM_032047 | 1.55983E-11 |
| NM_004776 | 0 |
| NM_001186 | 2.55791E-05 |
| NM_006317 | 1.11022E-16 |
| NM_006399 | 0 |
| NM_182648 | 4.62963E-14 |
| NM_004049 | 6.4122E-12 |
| NM_005178 | 0 |
| NM_003670 | 0 |
| AL133599 | 1.3981E-12 |
| NM_001725 | 2.00441E-07 |
| NM_144653 | 0 |
| NM_024850 | 5.84033E-12 |
| NM_004053 | 0 |
| NM_007021 | 1.53444E-12 |
| NM_173554 | 2.46417E-09 |
| NM_025113 | 4.75443E-09 |

| | |
|---|---|
| Contig37037_RC | 2.54241E-14 |
| NM_032245 | 0 |
| NM_030806 | 5.60663E-14 |
| NM_022083 | 2.55908E-05 |
| NM_004848 | 4.79471E-08 |
| NM_030945 | 3.33067E-16 |
| NM_018152 | 1.11022E-16 |
| NM_178817 | 2.22045E-16 |
| NM_144629 | 0 |
| NM_022912 | 0 |
| NM_000592 | 0 |
| NM_145316 | 1.01285E-10 |
| NM_181714 | 1.24356E-12 |
| NM_153362 | 5.55112E-16 |
| Contig47220 | 4.151E-08 |
| NM_005181 | 8.82678E-09 |
| X02330 | 1.30095E-09 |
| NM_005795 | 0 |
| NM_022162 | 7.86383E-11 |
| NM_004347 | 4.44611E-12 |
| NM_182511 | 4.9217E-11 |
| NM_002986 | 4.511E-08 |
| NM_002988 | 1.84154E-06 |
| NM_002982 | 0 |
| NM_002983 | 0 |
| NM_002984 | 1.43381E-07 |
| NM_006273 | 0 |
| NM_005623 | 0 |
| NM_020307 | 8.34114E-05 |
| NM_001295 | 0 |
| NM_003965 | 0 |
| NM_000591 | 3.62044E-13 |
| NM_001773 | 1.0949E-12 |
| NM_003503 | 5.18075E-10 |
| NM_022842 | 4.44089E-16 |
| NM_000389 | 0 |
| NM_001262 | 7.61613E-14 |
| NM_021146 | 2.32037E-14 |
| NM_001712 | 0 |
| NM_005195 | 0 |
| NM_003956 | 0 |
| NM_015424 | 0 |
| NM_014918 | 9.10383E-15 |
| NM_013324 | 1.64902E-06 |
| NM_144673 | 1.44329E-15 |
| NM_013246 | 5.21805E-15 |
| NM_005602 | 0 |
| NM_022570 | 0.000568026 |
| NM_013252 | 2.98827E-11 |
| NM_007261 | 8.70815E-06 |
| NM_000088 | 9.39233E-06 |
| NM_152890 | 0 |
| NM_032161 | 4.30378E-08 |
| NM_033427 | 2.38132E-12 |
| NM_000573 | 1.11246E-07 |
| AK021493 | 3.86691E-13 |
| NM_001881 | 1.23124E-13 |
| NM_001882 | 4.49175E-08 |
| NM_000758 | 9.59849E-11 |
| NM_000760 | 1.11022E-16 |
| NM_004385 | 1.70192E-10 |
| NM_024792 | 0 |
| NM_001905 | 0 |
| NM_001912 | 0 |
| NM_001511 | 0 |

| | |
|---|---|
| NM_002089 | 0 |
| NM_002090 | 1.6283E-05 |
| AB051463 | 1.03677E-11 |
| NM_000104 | 9.27948E-07 |
| NM_023944 | 2.22045E-16 |
| NM_001554 | 0 |
| Contig40298_RC | 9.32587E-15 |
| NM_080759 | 7.80999E-11 |
| AF318336 | 0 |
| NM_000574 | 1.02147E-08 |
| Contig8156_RC | 1.07137E-07 |
| NM_178152 | 4.35418E-12 |
| NM_032636 | 1.11022E-15 |
| NM_019058 | 0 |
| NM_004728 | 0 |
| NM_022779 | 0 |
| NM_004084 | 0.000139226 |
| NM_005217 | 1.30167E-09 |
| NM_022783 | 0 |
| NM_031476 | 0 |
| NM_030817 | 0 |
| AK025205 | 1.11022E-16 |
| NM_032297 | 0 |
| NM_013253 | 4.38538E-14 |
| NM_015569 | 0 |
| NM_001945 | 4.50751E-14 |
| NM_004417 | 1.60938E-06 |
| NM_004418 | 0 |
| NM_001394 | 0 |
| NM_057158 | 0 |
| NM_001946 | 9.63765E-09 |
| NM_032411 | 4.44089E-16 |
| Contig45618_RC | 3.08698E-08 |
| NM_001955 | 1.77248E-11 |
| NM_001964 | 0 |
| NM_000399 | 3.10862E-15 |
| NM_004430 | 2.22045E-16 |
| NM_152310 | 1.27126E-07 |
| NM_152939 | 1.32182E-08 |
| AL137578 | 7.47002E-12 |
| ENST00000274652 | 0.000887177 |
| ENST00000278205 | 4.22662E-13 |
| NM_005797 | 0 |
| NM_024582 | 2.62502E-07 |
| NM_058229 | 0 |
| NM_002001 | 4.44089E-16 |
| NM_000566 | 6.14428E-10 |
| NM_021642 | 1.55364E-05 |
| NM_031935 | 8.5703E-11 |
| NM_054014 | 0 |
| NM_004118 | 7.75142E-08 |
| NM_018059 | 0 |
| Contig37439_RC | 1.80274E-06 |
| NM_018349 | 1.20807E-08 |
| NM_022767 | 0 |
| NM_024709 | 1.77862E-11 |
| Contig56160_RC | 0 |
| NM_032849 | 1.14353E-14 |
| NM_032213 | 4.00588E-07 |
| Contig29921_RC | 5.27272E-10 |
| NM_024730 | 3.10269E-08 |
| NM_022837 | 3.33067E-16 |
| NM_024636 | 3.18624E-10 |
| NM_024616 | 1.12577E-12 |
| NM_024574 | 5.65914E-10 |

| | |
|---|---|
| NM_025079 | 0 |
| NM_024530 | 0 |
| Contig23475_RC | 0 |
| NM_182573 | 1.89293E-12 |
| NM_152320 | 5.45798E-09 |
| NM_144669 | 0 |
| NM_152680 | 9.55735E-06 |
| NM_152502 | 2.29033E-07 |
| Contig43338_RC | 1.15939E-09 |
| NM_144649 | 2.41825E-05 |
| NM_152608 | 0 |
| NM_175884 | 8.54872E-15 |
| Contig53242_RC | 2.78149E-07 |
| NM_152665 | 2.94863E-07 |
| NM_153690 | 3.33067E-16 |
| NM_153692 | 5.81942E-11 |
| NM_013231 | 6.69464E-14 |
| AB058769 | 4.60195E-07 |
| NM_022823 | 0 |
| NM_153756 | 9.65894E-15 |
| NM_005252 | 1.5527E-06 |
| NM_002029 | 0 |
| NM_174938 | 2.54241E-14 |
| NM_015440 | 1.11022E-16 |
| NM_015675 | 5.9619E-14 |
| NM_022134 | 1.16673E-12 |
| NM_024637 | 0 |
| NM_014863 | 2.47491E-11 |
| NM_015892 | 9.11493E-14 |
| NM_005257 | 1.25919E-07 |
| NM_001486 | 0 |
| NM_004864 | 0 |
| NM_005110 | 0.000561411 |
| NM_017655 | 0 |
| Contig40478_RC | 7.14617E-12 |
| Contig5961_RC | 1.05471E-14 |
| NM_002069 | 2.19824E-14 |
| NM_001505 | 1.44329E-15 |
| NM_005300 | 2.89856E-06 |
| NM_005282 | 0 |
| NM_005304 | 0 |
| NM_005310 | 1.76581E-07 |
| NM_173849 | 7.78266E-13 |
| NM_000854 | 1.96453E-08 |
| NM_016315 | 2.58815E-08 |
| NM_002108 | 1.47654E-05 |
| NM_021175 | 9.45455E-12 |
| NM_178232 | 1.11022E-16 |
| NM_001523 | 0 |
| NM_002110 | 3.98744E-07 |
| NM_012258 | 1.401E-09 |
| NM_153236 | 4.16334E-14 |
| Contig40128_RC | 0 |
| NM_001530 | 2.52842E-12 |
| NM_181054 | 0 |
| NM_022462 | 3.08133E-09 |
| NM_145899 | 5.7454E-13 |
| AF131827 | 0 |
| NM_002133 | 0 |
| NM_004503 | 1.63759E-10 |
| NM_153693 | 1.144E-08 |
| Contig53965_RC | 6.0407E-10 |
| NM_000196 | 5.78971E-11 |
| NM_016391 | 0 |
| NM_006644 | 7.03326E-13 |

| | |
|---|---|
| AJ227898 | 0 |
| AJ276240 | 8.68083E-13 |
| NM_153341 | 1.11022E-16 |
| NM_004907 | 0 |
| NM_003897 | 0 |
| NM_005531 | 0 |
| NM_006332 | 1.37411E-10 |
| NM_000618 | 5.30687E-14 |
| L27560 | 0 |
| NM_000599 | 2.72276E-10 |
| NM_178822 | 1.66655E-10 |
| NM_005849 | 8.79086E-08 |
| NM_054111 | 4.14446E-05 |
| NM_000572 | 0 |
| NM_002188 | 0 |
| NM_003855 | 7.23865E-14 |
| NM_003853 | 8.28734E-11 |
| NM_000576 | 0 |
| NM_004633 | 6.99668E-05 |
| NM_000577 | 1.26976E-12 |
| NM_014432 | 0.000138982 |
| NM_000418 | 0 |
| NM_000600 | 0 |
| NM_000584 | 0 |
| NM_001557 | 6.5592E-13 |
| NM_002193 | 1.11022E-15 |
| NM_005542 | 0 |
| NM_002198 | 0 |
| NM_002201 | 0 |
| NM_002205 | 0 |
| NM_000887 | 3.55271E-15 |
| Contig55048_RC | 0 |
| AB002344 | 0 |
| NM_002229 | 0 |
| NM_030929 | 0 |
| AB067508 | 6.31273E-13 |
| NM_002243 | 0 |
| NM_170736 | 1.11022E-16 |
| NM_000891 | 1.13706E-10 |
| NM_003740 | 0 |
| NM_014734 | 2.38025E-10 |
| NM_015179 | 0 |
| NM_014963 | 0 |
| AB033065 | 5.94173E-07 |
| AB037766 | 3.92824E-11 |
| AB040938 | 0 |
| AB051458 | 0 |
| NM_032550 | 0 |
| NM_032873 | 5.60414E-08 |
| NM_014079 | 2.211E-07 |
| NM_130446 | 2.97775E-08 |
| NM_005559 | 0 |
| NM_005565 | 0 |
| NM_000527 | 0 |
| NM_002309 | 2.00375E-11 |
| NM_006864 | 0 |
| NM_016733 | 1.11022E-16 |
| NM_005569 | 1.11022E-16 |
| NM_006033 | 3.36794E-10 |
| NM_021250 | 0 |
| NM_004862 | 0 |
| NM_002315 | 1.11022E-16 |
| NM_052879 | 0 |
| Contig38772_RC | 1.11022E-16 |
| NM_178833 | 0 |

| | |
|---|---|
| NM_144697 | 0 |
| NM_145282 | 8.5407E-10 |
| NM_174918 | 0 |
| NM_175903 | 1.78501E-07 |
| Contig63026 | 0 |
| AW673036_RC | 6.56514E-11 |
| ENST00000295549 | 0 |
| Contig4595 | 0 |
| NM_016644 | 0 |
| NM_018687 | 1.84131E-08 |
| NM_020143 | 3.87681E-07 |
| NM_030802 | 0 |
| NM_053040 | 0 |
| NM_138779 | 1.76759E-12 |
| NM_015236 | 6.51701E-14 |
| NM_052972 | 0 |
| NM_181726 | 0 |
| NM_018334 | 0 |
| NM_032808 | 1.12133E-14 |
| NM_005584 | 4.12754E-11 |
| Contig46202 | 1.44351E-09 |
| NM_012323 | 0 |
| NM_030801 | 0 |
| NM_031419 | 0 |
| NM_005906 | 1.09896E-09 |
| NM_052886 | 1.00928E-11 |
| NM_153267 | 3.72869E-09 |
| NM_005204 | 7.13789E-08 |
| AK054569 | 0 |
| NM_005911 | 0 |
| NM_182763 | 0 |
| NM_005924 | 1.50468E-08 |
| NM_138395 | 0 |
| NM_052943 | 8.0883E-10 |
| NM_152421 | 1.22125E-14 |
| NM_144683 | 2.43024E-10 |
| NM_152430 | 2.22045E-15 |
| NM_152339 | 0 |
| NM_152435 | 7.83273E-12 |
| Contig29982_RC | 3.77476E-15 |
| NM_052871 | 1.51615E-11 |
| AL512725 | 0 |
| NM_032390 | 0 |
| NM_024101 | 5.18685E-12 |
| NM_022468 | 3.33067E-16 |
| NM_022718 | 6.4837E-14 |
| NM_022122 | 2.6701E-07 |
| NM_002422 | 0 |
| NM_005098 | 5.24913E-13 |
| NM_005946 | 0 |
| NM_005951 | 1.8743E-10 |
| NM_175622 | 0 |
| NM_176870 | 0 |
| NM_002450 | 3.33067E-16 |
| NM_005952 | 2.19158E-13 |
| NM_005953 | 6.66134E-15 |
| NM_006636 | 9.50906E-13 |
| NM_138299 | 2.3237E-13 |
| NM_002463 | 1.64602E-12 |
| NM_000256 | 4.27742E-10 |
| NM_004997 | 3.21965E-15 |
| NM_002467 | 0 |
| NM_002474 | 8.46735E-05 |
| AK025953 | 3.83027E-14 |
| NM_012335 | 5.62395E-10 |

| | |
|---|---|
| AK054570 | 0 |
| NM_000433 | 5.74762E-12 |
| NM_014397 | 0 |
| NM_080741 | 0 |
| NM_002502 | 4.21885E-15 |
| NM_006167 | 0 |
| NM_015039 | 0 |
| NM_006169 | 0 |
| NM_006170 | 0 |
| NM_006392 | 0 |
| NM_004741 | 0 |
| NM_020962 | 8.88178E-15 |
| NM_000270 | 0 |
| NM_002523 | 6.66134E-16 |
| NM_000910 | 5.64215E-05 |
| NM_002135 | 0 |
| NM_173198 | 0 |
| NM_004883 | 1.44329E-15 |
| NM_013981 | 6.65024E-14 |
| NM_013982 | 1.86406E-13 |
| NM_014366 | 7.69385E-14 |
| NM_021229 | 6.66134E-16 |
| NM_139131 | 0 |
| NM_014778 | 5.24025E-14 |
| NM_007225 | 6.15294E-05 |
| NM_013248 | 0 |
| NM_014279 | 2.9976E-15 |
| NM_020190 | 2.97651E-13 |
| NM_020530 | 0 |
| NM_053001 | 0 |
| NM_012387 | 1.43683E-10 |
| Contig51888_RC | 4.07249E-06 |
| NM_015368 | 1.11022E-16 |
| NM_005746 | 1.34004E-13 |
| NM_182790 | 4.97924E-09 |
| AL137559 | 0 |
| NM_000439 | 1.412E-09 |
| NM_021830 | 0 |
| NM_002616 | 0 |
| NM_004427 | 0 |
| AK026181 | 5.56444E-13 |
| Contig54031 | 1.67533E-13 |
| Contig53952_RC | 9.17596E-05 |
| NM_012417 | 6.90619E-07 |
| Contig43433_RC | 1.0206E-08 |
| NM_022062 | 9.97991E-12 |
| NM_000299 | 1.12002E-11 |
| NM_024420 | 1.07703E-11 |
| NM_000929 | 0 |
| NM_000930 | 3.88578E-15 |
| NM_002658 | 2.72112E-11 |
| AB033035 | 3.46443E-06 |
| NM_004073 | 0 |
| NM_021105 | 5.556E-08 |
| NM_021127 | 7.78266E-12 |
| NM_015029 | 1.22125E-15 |
| NM_002705 | 3.9968E-15 |
| NM_014330 | 3.41949E-14 |
| NM_015062 | 0 |
| NM_020820 | 4.77182E-11 |
| NM_002727 | 1.74768E-08 |
| AK055479 | 0 |
| NM_021935 | 0 |
| NM_017456 | 9.99201E-16 |
| NM_130435 | 5.87921E-11 |

| | |
|---|---|
| NM_006504 | 9.02171E-08 |
| NM_002852 | 0 |
| NM_025215 | 0 |
| NM_006505 | 0 |
| NM_017817 | 1.11022E-16 |
| AK055564 | 0 |
| NM_003979 | 0 |
| NM_000964 | 0 |
| NM_170774 | 4.39648E-14 |
| NM_031437 | 7.3797E-08 |
| NM_006509 | 0 |
| NM_032918 | 1.6323E-07 |
| NM_153615 | 3.096E-11 |
| NM_002928 | 0 |
| NM_002923 | 9.66038E-12 |
| NM_015444 | 0 |
| NM_024787 | 0 |
| NM_024778 | 0 |
| NM_173647 | 8.87864E-11 |
| NM_015169 | 0 |
| NM_001754 | 0 |
| NM_002964 | 0 |
| NM_002965 | 2.03682E-12 |
| NM_005980 | 1.44329E-15 |
| NM_030754 | 1.98308E-12 |
| NM_022136 | 0.000544437 |
| NM_178135 | 8.09131E-13 |
| NM_006998 | 2.22045E-16 |
| NM_003004 | 0 |
| NM_000450 | 8.42981E-12 |
| NM_000655 | 8.17131E-09 |
| NM_004186 | 0 |
| NM_017789 | 1.56541E-14 |
| NM_030666 | 1.71648E-11 |
| NM_002575 | 2.26969E-07 |
| NM_002640 | 0 |
| NM_000602 | 9.32576E-12 |
| NM_005025 | 4.70287E-09 |
| NM_013376 | 0 |
| NM_019605 | 5.25013E-12 |
| NM_005627 | 6.03595E-12 |
| Contig60194_RC | 0 |
| NM_173216 | 0 |
| NM_018414 | 2.66454E-15 |
| NM_152996 | 0 |
| NM_005982 | 2.2282E-11 |
| NM_006748 | 9.6099E-05 |
| NM_004207 | 0 |
| NM_006996 | 3.87701E-09 |
| NM_007105 | 0 |
| NM_003059 | 3.33746E-06 |
| AK057476 | 2.22045E-16 |
| AB067483 | 2.22045E-16 |
| NM_153449 | 3.55271E-15 |
| NM_006931 | 0 |
| NM_003039 | 1.6477E-11 |
| D31887 | 2.97196E-12 |
| NM_003615 | 0 |
| Contig56768_RC | 6.02264E-07 |
| AL050021 | 0 |
| NM_003486 | 0 |
| NM_016354 | 9.84492E-10 |
| NM_030952 | 1.34115E-13 |
| NM_173354 | 0 |
| NM_018664 | 0 |

| | |
|---|---|
| NM_003498 | 0 |
| NM_000636 | 0 |
| Contig40954_RC | 0.000128792 |
| NM_031439 | 1.48065E-11 |
| NM_021972 | 0 |
| NM_152594 | 3.11071E-08 |
| NM_080862 | 9.41669E-10 |
| AB037721 | 0 |
| NM_004760 | 1.11022E-16 |
| NM_014465 | 3.74272E-07 |
| NM_004711 | 1.02141E-14 |
| NM_006474 | 0 |
| NM_052864 | 2.69127E-10 |
| NM_032505 | 0 |
| NM_015472 | 1.09024E-12 |
| NM_018421 | 0 |
| NM_003213 | 0 |
| NM_003236 | 0 |
| NM_000361 | 2.26146E-10 |
| NM_013390 | 2.37588E-14 |
| NM_002160 | 3.23592E-09 |
| NM_000594 | 0 |
| NM_007115 | 1.12759E-07 |
| NM_147187 | 2.22045E-16 |
| NM_003841 | 5.63993E-14 |
| NM_001065 | 0 |
| NM_022144 | 4.60021E-10 |
| NM_006114 | 3.33067E-16 |
| Z36778 | 0 |
| NM_018643 | 0 |
| NM_174892 | 6.30344E-05 |
| NM_025195 | 0 |
| NM_021158 | 0 |
| NM_178125 | 3.14193E-14 |
| NM_005725 | 1.44329E-15 |
| NM_133378 | 7.73825E-14 |
| NM_006086 | 1.79967E-13 |
| NM_006472 | 1.82632E-13 |
| NM_003330 | 0 |
| NM_018003 | 0 |
| Contig47339_RC | 0 |
| NM_012474 | 0 |
| NM_181597 | 4.44089E-16 |
| NM_031471 | 4.59628E-11 |
| Contig42760_RC | 0 |
| NM_025090 | 0 |
| NM_003370 | 0 |
| NM_000376 | 0 |
| NM_052958 | 3.77476E-15 |
| NM_004665 | 1.11022E-15 |
| NM_018399 | 3.75133E-09 |
| NM_000377 | 5.25637E-08 |
| NM_003391 | 4.01482E-06 |
| NM_006691 | 1.44174E-12 |
| NM_006006 | 3.33067E-16 |
| Contig43253_RC | 1.06398E-10 |
| NM_032772 | 3.88967E-12 |
| NM_015461 | 1.86517E-14 |
| Contig2728_RC | 1.38457E-08 |
| Contig51292_RC | 0 |
| Contig31872_RC | 2.65277E-09 |
| Contig21997_RC | 0 |
| Contig43708_RC | 1.69644E-11 |
| Contig13480_RC | 0 |
| Contig47512_RC | 0 |

| | |
|---|---|
| Contig50728_RC | 7.74316E-09 |
| Contig37690_RC | 1.11428E-07 |
| Contig26835_RC | 2.35026E-11 |
| Contig35400_RC | 0 |
| Contig23085_RC | 0 |
| Contig48806_RC | 0 |
| Contig32336_RC | 9.66181E-05 |
| Contig40785_RC | 0 |
| Contig23198_RC | 0 |
| Contig22534_RC | 4.68815E-09 |
| Contig36902_RC | 0 |
| Contig35897_RC | 0 |
| Contig24282_RC | 1.74422E-11 |
| Contig28286_RC | 0 |
| AL080082 | 1.48826E-07 |
| AK000802 | 0 |
| AL049337 | 8.88178E-16 |
| Contig48076_RC | 1.40155E-12 |
| AI401061_RC | 0 |
| Contig37538_RC | 1.54168E-08 |
| Contig36931_RC | 6.66134E-16 |
