# Supplementary material for: Diurnal variation of the human adipose transcriptome and the link to metabolic disease
Source: BMC Med Genomics. 2009 Feb 9;2:7. doi: 10.1186/1755-8794-2-7 (PMC2647943; doi:10.1186/1755-8794-2-7)
Supplement: Additional file 6 — Transcripts that are significantly correlated with PER1 probe. Absolute value of Spearman correlation coefficient higher than 0.4. [file 1755-8794-2-7-S6.pdf]

Transcript	Correlation to Per1
NM_023037	0.408083636
AK001838	-0.572535531
NM_020772	-0.741224811
NM_153332	-0.562009813
NM_017436	-0.444963267
NM_004920	-0.493418772
NM_080282	0.542455342
NM_001089	0.410605513
NM_172346	0.556198643
NM_019625	0.539509281
NM_033450	0.545205097
NM_000392	0.647959395
NM_002940	-0.687542895
NM_001090	-0.550194366
NM_016818	-0.715391501
NM_152924	-0.429498833
NM_005157	-0.487207421
Contig56303_RC	-0.637996405
NM_005158	-0.638564934
NM_013375	-0.550926694
NM_145804	-0.422543399
NM_024722	0.441745917
NM_020039	0.452675887
NM_003500	0.483524968
NM_004457	-0.477269981
NM_004458	-0.588761821
NM_022977	-0.643448221
NM_001101	-0.469445348
NM_001614	-0.507202692
NM_005721	-0.468229964
NM_004302	-0.479185327
NM_001616	-0.474187583
NM_001106	0.520197712
NM_001107	0.410242348
NM_014064	-0.514674581
NM_033274	-0.525316499
NM_001109	-0.424778254
NM_006988	-0.560592308
NM_139025	0.471990666
NM_014243	-0.498874437
NM_020249	-0.662287873
NM_182920	-0.715371066
NM_015834	0.456774569
NM_014190	0.475383084
NM_016824	0.414863586
NM_001122	-0.427430384
NM_001124	-0.636740791
NM_017825	-0.670133272
NM_032264	0.433668706
AF075057	0.567318962
AF085900	0.451565889
AF086149	-0.625864437
AF086164	-0.487396735
AF086261	0.545757588
AF147300	-0.46469556
AF147344	0.545193744
AF333388	-0.514837058
NM_024524	-0.66004679
NM_004835	0.478671944
NM_152392	0.440900729
AK000839	0.545122776
AK001060	0.626331216
AK001139	0.475750391
AK001808	0.512523569

AK021439	0.565068999
AK022092	0.529185691
AK022095	0.420782124
AK022482	0.476637944
AK022635	0.410038401
AK023526	0.668757202
AK023565	0.413538029
AK024118	0.458225623
AK024385	-0.530457435
AK024545	0.432064648
AK024944	-0.545030888
AK025246	0.407586785
AK054959	0.645670054
AK054971	0.551594322
AK054990	0.590649794
AK055014	0.50897941
AK055553	0.484850285
AK056146	0.517345555
AK056228	0.725185992
AK057578	0.663245322
AK057759	0.567113221
AK057835	0.424980379
NM_005858	-0.45565019
AL050004	-0.430480502
AL512701	0.463551773
NM_000693	-0.464299644
NM_000034	0.624380933
NM_024105	0.428294593
NM_033087	-0.54338641
NM_006020	-0.625347599
Contig47930_RC	0.411449133
NM_173511	0.432930047
NM_001634	-0.742481164
NM_020547	0.415039758
NM_001145	0.427289974
NM_001146	0.412063819
NM_001147	-0.447997647
NM_139314	-0.460335975
NM_024668	-0.545323376
NM_032217	-0.430479796
NM_144994	0.581840292
NM_173505	0.461462258
NM_001128	-0.446110966
NM_001640	0.502661069
NM_014481	-0.465739977
NM_030803	-0.650605555
NM_017413	-0.499122164
NM_145699	-0.484246171
NM_145637	-0.450199363
NM_030882	-0.45963231
NM_020979	-0.616991412
NM_001657	-0.558281009
NM_021069	0.651349009
NM_004308	0.41341266
AF038193	0.496222136
NM_178815	-0.640614998
NM_030978	-0.623465778
NM_001670	0.430595145
Contig44538_RC	0.494654082
NM_017873	-0.407857391
NM_024708	-0.442984219
NM_024087	0.440045877
NM_006716	-0.472894767
NM_133436	-0.574865221
NM_018188	-0.459521008

NM_006885	0.44393629
NM_004024	-0.643642701
NM_001674	-0.663444761
NM_020749	0.507874933
NM_032827	0.625865117
NM_001679	-0.577871342
NM_170665	-0.651066602
NM_012463	-0.49575884
NM_001693	-0.58180365
NM_144583	0.578070044
NM_004888	-0.502543081
AK057452	0.407958146
NM_178190	0.536435044
NM_021732	0.474175415
NM_000054	0.464957941
NM_181050	-0.430538047
NM_001699	0.43923201
NM_033027	-0.538339129
AB029041	0.531865721
NM_003782	0.400501073
NM_006577	-0.427196652
NM_032047	-0.571614764
NM_003779	-0.691083441
NM_004776	-0.779950489
NM_001186	-0.474988707
NM_004281	-0.685690364
NM_004873	-0.411038555
NM_014952	-0.452487847
NM_003933	0.542245983
NM_006317	-0.487959344
NM_033177	0.480302229
NM_025256	0.525604425
NM_006399	-0.627962125
NM_013448	-0.534874338
NM_182648	-0.671769381
NM_024649	0.447143766
NM_020235	0.453894783
NM_021948	0.449485956
NM_014567	-0.558574554
NM_078468	-0.524006079
NM_005178	-0.71914227
Contig53619_RC	-0.500276583
NM_004326	0.47750196
NM_000710	-0.405160243
NM_000623	-0.425705032
NM_005434	-0.430600004
NM_001195	0.533157932
NM_003670	-0.584298276
Contig26622_RC	-0.486338675
NM_139346	0.408342147
NM_016077	-0.793207875
NM_018455	-0.521378142
AL133599	0.476644712
NM_033254	0.443974424
NM_018321	-0.719287293
NM_015399	-0.45574617
NM_017942	-0.498334324
NM_144653	-0.669097755
NM_017797	0.456757082
NM_000060	0.485368706
NM_007048	0.564376828
AK056264	-0.572678775
NM_004053	-0.765570326
NM_007021	-0.411996092
NM_153244	-0.537782559

NM_022451	-0.638860168
NM_024834	-0.54453213
NM_032804	-0.652225115
NM_032709	0.415052058
NM_018464	-0.549413208
AL080114	0.470220401
NM_020644	-0.584226137
NM_021238	-0.457799365
AL122049	-0.537503808
NM_030809	-0.615068784
NM_020374	-0.505510327
AK000954	-0.432049754
NM_025113	-0.427803766
Contig37037_RC	-0.672162814
NM_032859	-0.432854633
Contig3612_RC	-0.42612934
NM_018139	-0.560794605
NM_018453	0.430705039
NM_020215	0.477098947
NM_022067	0.528933207
NM_024558	-0.408874163
NM_024644	-0.54476189
NM_152330	-0.457815197
NM_144578	-0.533070916
AK000671	0.476671033
NM_021944	0.685065611
NM_017815	0.498039551
NM_030630	0.658941701
NM_152260	-0.427511987
NM_152352	-0.677484688
NM_024805	0.483956027
NM_013326	-0.409875896
NM_032245	-0.633493202
NM_152474	0.40794599
NM_030806	0.480206755
NM_016183	-0.492541111
NM_024319	0.407922056
NM_001212	-0.507224206
NM_012072	-0.40060868
NM_030945	0.50333805
NM_031911	0.407384604
NM_080821	0.439226235
NM_080725	-0.481496014
NM_152667	-0.576019624
NM_152302	-0.634530857
NM_018270	-0.419448141
NM_017798	-0.651164216
NM_018257	0.401952218
NM_015511	-0.437850514
NM_032957	-0.507804324
NM_018354	0.470507343
NM_178472	-0.505517775
NM_016649	-0.636828699
NM_032653	0.476856191
NM_017438	0.42917489
NM_178817	0.448756027
NM_058187	-0.521749361
NM_144629	0.489143967
NM_138611	-0.591080757
NM_022912	0.549072493
NM_015680	0.517760171
NM_017546	-0.529821287
NM_005768	-0.471007699
NM_000592	-0.637138318
NM_016348	0.621257206

NM_015388	0.467984265
NM_005452	-0.436060639
NM_033069	-0.401697207
NM_031452	-0.41823432
NM_145316	-0.446638584
Contig57908_RC	-0.474225708
NM_153362	0.40916696
NM_019052	0.415539006
NM_030651	0.511806088
NM_014165	-0.533633393
NM_018247	-0.536167613
NM_138459	-0.481770661
AK002014	0.440482052
NM_015439	0.414760112
Contig55114_RC	0.414431859
NM_032860	-0.447472996
NM_024051	-0.613930252
NM_138446	-0.440581682
AK024443	0.444210362
NM_022755	-0.674498493
NM_016446	0.404075461
NM_144966	0.454344225
AK000001	0.49296992
Contig49409	-0.497021687
NM_031426	0.516117885
NM_006336	0.485724798
Contig47220	-0.553375151
Contig6610_RC	-0.547810843
X02330	-0.543015327
NM_005795	0.449938095
NM_003656	0.467994239
Contig20908_RC	0.471630481
NM_001222	0.432156672
NM_005186	0.508787892
NM_173087	0.491749956
NM_004055	0.418085755
NM_080590	0.466775361
NM_014550	0.465175689
NM_022162	-0.422486883
NM_139273	-0.428051562
NM_032976	0.492821717
NM_001225	-0.531506337
NM_004347	-0.431433331
NM_001226	0.47284778
NM_033338	-0.513468187
NM_004349	0.430434702
NM_005187	0.433067243
NM_001755	-0.521590176
NM_182511	0.433045581
NM_000071	-0.579025005
NM_175709	0.498833179
NM_004059	0.639755568
NM_002986	-0.519773784
NM_002982	-0.699940386
NM_002983	-0.475416346
NM_006273	-0.60022073
NM_005623	-0.743610545
AK000799	-0.536610237
NM_020307	-0.445399845
NM_001295	-0.528526008
NM_003965	-0.698844558
NM_006431	-0.657390283
NM_005998	-0.411884015
NM_012073	-0.402514069
NM_000591	-0.425410289

NM_001766	-0.426051183
NM_001773	0.613890664
NM_000611	-0.471204566
NM_004233	-0.445478299
NM_002414	0.476463834
Contig28947_RC	-0.421168821
NM_006779	-0.598179313
NM_001254	-0.546127779
NM_003503	-0.575257576
NM_022842	-0.540960913
NM_052827	-0.58365631
NM_000389	-0.703362004
NM_000076	0.476409527
M63256	-0.496652897
NM_021146	0.436767402
NM_001712	-0.722721272
NM_005194	-0.47727335
NM_005195	-0.553413433
NM_001806	-0.499360327
NM_005760	-0.576826653
NM_001408	0.414784574
NM_015230	-0.46974006
NM_007018	0.477622008
NM_015939	-0.644038234
NM_016052	-0.675640652
NM_015942	-0.544184968
NM_016101	-0.695357405
NM_016140	0.479203735
NM_015964	0.475064387
NM_016001	-0.715523382
NM_032205	-0.438460021
NM_003956	-0.714677449
NM_001269	-0.558699186
NM_001270	-0.548405876
NM_006387	-0.490338117
NM_018223	-0.426331618
NM_012124	-0.643351117
NM_015424	-0.728458653
NM_004854	-0.594196485
NM_004267	-0.419213291
NM_014918	-0.659552951
NM_001278	-0.491705372
NM_032830	-0.650905586
NM_016274	-0.464384762
NM_138410	-0.533250854
NM_013246	-0.568775551
NM_005602	0.400358232
NM_001305	0.433380539
NM_013252	-0.44943922
NM_001292	0.418692535
NM_014944	0.644797298
NM_030629	-0.416006964
NM_020311	-0.516497938
NM_032869	-0.459846957
NM_014865	0.511280213
Contig27972_RC	0.477406511
NM_018366	-0.615875587
NM_016083	0.476757689
NM_015386	0.552576797
NM_032382	-0.52170907
NM_030582	0.483352073
NM_173465	0.466789012
NM_152890	0.47940872
NM_032161	-0.504572632
NM_080541	0.674309954

AK000009	0.409600179
NM_000754	0.406415683
NM_007310	0.403515992
NM_022730	0.460752546
NM_014325	-0.492729909
NM_001302	0.470905897
NM_030627	-0.479176962
NM_152727	0.407087409
NM_020939	-0.483735388
NM_153634	-0.557289995
NM_006693	-0.454631637
NM_007007	-0.512432559
Contig41560_RC	0.497650778
NM_152359	0.465714427
NM_130898	0.497453603
NM_001881	-0.582342948
NM_016507	-0.46957058
NM_014675	0.513442812
NM_004269	-0.430569136
NM_145311	0.514582024
NM_015989	0.473156198
NM_013434	0.426937732
NM_000758	-0.54308107
NM_001893	-0.464888375
Contig54259_RC	-0.469524622
NM_004385	-0.428459026
NM_175856	-0.403701555
NM_015235	-0.61581253
NM_024792	-0.693563744
NM_020428	0.468389031
NM_001905	-0.781112654
NM_001912	-0.732336237
NM_003592	-0.423386312
NM_014780	0.557051459
NM_181500	0.42149008
NM_001511	-0.694697769
NM_004887	0.46620769
NM_002089	-0.724571729
NM_005491	-0.412472213
NM_025212	0.51090276
NM_000103	-0.45159435
NM_000104	-0.484379201
NM_023944	0.547573141
NM_000786	-0.503588161
NM_001554	-0.719990822
NM_015345	0.460931373
Contig2930_RC	-0.591141312
NM_001343	-0.458984615
AF318336	0.523281186
NM_000574	-0.489352396
NM_020188	-0.430978561
Contig8156_RC	0.407595692
NM_032636	0.608918455
NM_012137	-0.574381037
NM_000107	0.536361791
NM_004083	-0.446201074
NM_019058	0.55373092
NM_004398	-0.619837076
NM_006773	-0.550868474
NM_007204	-0.413423181
NM_004728	-0.732162118
NM_012141	-0.497353112
NM_018380	-0.59377262
NM_022779	-0.683949303
NM_005804	-0.669890996

NM_001356	-0.494022821
NM_016355	-0.506907444
NM_014740	-0.640784665
AL133585	-0.467912204
NM_007010	-0.424351582
NM_152300	-0.428653773
NM_032998	-0.455879026
NM_022783	0.459881089
Contig569_RC	-0.579793846
L77565	-0.592403705
NM_178009	-0.450687069
NM_001358	-0.460182881
NM_014966	-0.47943805
Contig53750_RC	-0.733045755
NM_020162	-0.564589367
NM_032656	-0.55000222
NM_000398	0.421856951
NM_007326	-0.502101519
NM_022345	0.582635083
NM_018982	-0.423041588
NM_001363	-0.538882642
NM_031476	-0.607630724
NM_015426	-0.603145612
NM_030817	-0.599259232
NM_032254	0.520284222
NM_015412	-0.593980133
NM_022742	0.523133228
NM_015492	-0.506971681
NM_017606	0.467273262
NM_152892	-0.4495906
NM_022778	-0.6305304
Contig55334_RC	0.561671146
NM_020161	-0.644681803
NM_032018	-0.505386415
NM_015582	0.484675796
NM_015459	-0.4205897
AF074987	-0.538976471
NM_015420	-0.699940779
AK025205	0.445216987
Contig25362_RC	-0.431434382
NM_030797	-0.456100101
NM_015509	-0.490964297
NM_030923	0.465032413
NM_015533	0.517409452
NM_015462	-0.558140403
NM_015400	-0.60833491
Contig2313_RC	0.401658048
NM_177966	-0.783726954
NM_032297	0.524938767
RSE_00000563272	-0.430813068
NM_152661	0.460249374
NM_144996	-0.423363278
AK024495	0.432201377
Contig47102_RC	0.570309067
NM_178542	0.610513072
AL390216	0.559030517
NM_019100	0.409225844
NM_001539	-0.529768281
NM_005740	0.467991672
AF118274	0.672276662
NM_139072	0.438140473
NM_015569	0.508326125
NM_005851	0.472055988
NM_018431	-0.583565646
NM_001383	-0.423672913

NM_001384	-0.66014761
NM_001387	0.611885404
Contig54915_RC	0.412030429
AL137332	-0.492099085
NM_032581	-0.561783665
NM_014503	-0.655516453
NM_004414	-0.649643471
NM_003720	-0.593264946
NM_001945	-0.603734837
NM_020892	-0.408761954
NM_178502	0.490342177
NM_004418	-0.510933124
NM_057158	-0.651518839
NM_001394	-0.714714809
NM_004419	-0.51636988
NM_001946	-0.557353885
NM_001949	-0.440976394
NM_001951	-0.625510811
NM_001952	-0.519528911
NM_016565	-0.424083769
Contig37295_RC	-0.544677974
NM_024007	0.446877243
NM_024693	0.460741884
NM_032411	0.503614231
Contig52405_RC	-0.564142239
NM_001955	0.525696266
NM_025202	0.443332372
NM_024329	-0.418184074
NM_182685	0.45916936
NM_025205	-0.477039152
NM_001964	-0.729747691
NM_000399	-0.588143872
NM_004430	-0.683213829
NM_006795	-0.488147374
Contig176_RC	-0.445334068
NM_003758	-0.438005445
NM_003751	-0.481162284
NM_001416	-0.625956096
NM_001968	-0.465709099
NM_004846	-0.402940557
NM_018638	-0.413824815
NM_001421	-0.410929453
NM_005229	-0.566914041
NM_001973	-0.439773091
NM_012081	-0.439352033
NM_001423	-0.409181858
AL137578	0.462524398
ENST00000238062	-0.508530748
ENST00000253016	-0.578722776
ENST00000266926	-0.471535608
ENST00000272235	-0.43488439
ENST00000274966	-0.47731378
ENST00000277872	-0.484564969
ENST00000278205	-0.572973823
ENST00000282037	-0.580891983
ENST00000299111	-0.54812855
Contig30671_RC	0.50396727
NM_004431	-0.488109447
NM_001979	0.501766178
NM_014597	-0.525975546
NM_000124	0.475229867
NM_004451	-0.482303118
NM_004730	-0.547961253
NM_005239	-0.457824723
NM_001987	-0.494432454

NM_144765	-0.509802723
NM_005797	-0.725967602
NM_147127	0.426092111
NM_015219	0.466961948
NM_016042	-0.726466551
NM_019037	-0.428194129
NM_001991	0.716944028
Contig51006_RC	-0.625842852
NM_145249	0.425646749
NM_020223	-0.542536158
NM_022068	0.406415705
NM_021922	0.505618765
NM_004629	0.492743814
NM_006567	0.41606513
NM_004461	-0.52600647
NM_032145	-0.494592604
Contig56270_RC	0.463499175
AK023391	0.415869976
NM_058229	0.716990853
NM_000566	-0.432191386
NM_004462	0.442190219
NM_020482	0.494254946
NM_014344	-0.410540783
NM_054014	-0.62828171
NM_182827	0.438347881
NM_004118	0.428594499
NM_031307	-0.735408049
NM_031904	-0.571369603
NM_052847	0.681859116
NM_017991	0.431289884
NM_017994	-0.491335872
NM_018035	0.4823576
NM_018059	0.561942615
NM_018066	-0.539950287
NM_018072	-0.526868321
NM_032239	-0.643512486
NM_019057	0.461693085
NM_018089	0.582184177
NM_018093	-0.517475751
NM_018130	-0.599816418
NM_018134	0.490979415
NM_018138	-0.658675011
NM_024662	-0.520336395
NM_018233	-0.454719685
NM_018346	-0.565165781
NM_018370	-0.476002603
NM_018379	0.446472396
NM_018390	-0.401249466
NM_024666	-0.611124336
NM_024748	0.598365653
NM_024603	0.475908416
NM_021934	-0.656630881
NM_024891	0.494578669
NM_025155	0.458368906
NM_023077	-0.62529278
NM_022767	-0.765606971
NM_032168	-0.465860846
NM_023074	-0.466841701
NM_024998	-0.575635899
NM_024855	-0.462443914
NM_024742	-0.497196093
Contig58353_RC	-0.484355006
NM_025000	-0.409437728
NM_021826	-0.572577924
NM_024853	0.416525217

NM_025147	-0.556272129
NM_024706	-0.457315248
NM_024868	0.410391266
NM_024709	0.594267837
NM_024903	0.523619377
NM_025069	-0.509207991
NM_024733	0.418495091
Contig56160_RC	-0.691811545
NM_032849	-0.618116718
NM_032284	-0.594311148
NM_032866	0.506792366
NM_017622	0.481229965
NM_015590	-0.684006525
NM_017733	0.433423579
NM_019027	-0.450135441
NM_017755	-0.627419091
NM_019005	-0.472291203
NM_017776	0.500020542
NM_017777	0.445505731
NM_017782	-0.598081716
NM_017837	0.46235827
NM_017841	-0.457489694
NM_017860	-0.42190425
NM_017866	-0.563830607
NM_017888	0.405280116
NM_017908	0.549807273
NM_017916	0.530176049
Contig49512_RC	0.419141522
NM_017940	0.430055331
NM_017956	-0.43066137
NM_017966	-0.50781726
NM_024600	0.591580949
Contig773	-0.63078491
NM_024627	-0.482543235
NM_024549	0.536126102
NM_024602	0.500795209
NM_025032	0.406993627
NM_024738	-0.497698211
NM_021831	0.424822923
NM_023015	0.538380761
NM_032213	0.455648859
NM_024561	-0.51517209
NM_024836	-0.595805157
Contig36364_RC	-0.523224847
NM_024717	-0.640770173
NM_024697	-0.650130551
NM_025140	0.42748397
NM_024785	-0.426267881
NM_022074	0.408219809
NM_022837	-0.713682279
NM_024636	-0.569984759
NM_024616	-0.678025956
NM_025079	-0.580879566
NM_024530	-0.65141151
NM_024766	0.456798764
Contig23475_RC	-0.556903619
NM_024640	-0.649373037
NM_182500	0.436291123
NM_152546	-0.626903026
Contig13706_RC	0.413230929
NM_145018	-0.529139422
NM_153008	0.528930544
NM_174950	-0.50765609
NM_182573	-0.594272996
NM_153014	-0.533442663

NM_152461	-0.565099744
NM_144609	-0.489440085
NM_144726	-0.424186338
NM_144669	-0.426507708
NM_173795	0.4821064
NM_152502	0.457817347
Contig43338_RC	0.529681281
NM_152440	-0.521350029
NM_152415	-0.452319161
NM_152500	0.507924341
NM_175918	0.404417516
NM_152608	-0.598412795
Contig53242_RC	-0.537952977
NM_175884	-0.674470275
NM_173622	0.454200985
NM_152716	-0.55862234
Contig48021_RC	-0.473631789
NM_173507	0.408514624
NM_152382	-0.435746676
NM_153261	-0.40102247
NM_153689	-0.427056348
NM_178519	0.421865825
AF086471	-0.454752726
NM_152307	-0.520317001
NM_173677	0.424298483
NM_152665	-0.535837686
NM_153690	-0.491235858
NM_175919	0.560113143
NM_014053	-0.492984468
NM_022823	-0.625131022
NM_153756	0.609698218
NM_002028	0.45291978
NM_018416	0.513847306
NM_004514	-0.485233501
NM_002029	-0.464314526
NM_181355	0.435452348
NM_024919	0.453496926
NM_174938	0.452848548
NM_006653	0.49515164
NM_015440	-0.618660133
U79260	0.464968684
NM_003902	-0.577282588
NM_145059	0.468351678
NM_006625	-0.466485485
NM_000148	-0.414051922
NM_022003	0.501652209
NM_153047	0.600794888
NM_031866	-0.682132708
NM_005754	-0.468293002
NM_006360	-0.417719398
NM_016654	-0.410763067
NM_002041	-0.510224062
NM_001924	-0.430171021
NM_015675	-0.529235733
NM_024637	0.756359221
NM_000403	-0.406972413
NM_014863	-0.489788384
NM_015892	-0.505747442
NM_018590	-0.41381506
NM_020474	-0.550344545
NM_000155	0.665214126
NM_147131	0.478703996
NM_147132	0.463749787
NM_032293	0.559492379
NM_005512	-0.450402059

NM_002047	-0.547545938
NM_175085	-0.497888251
NM_005257	-0.597120882
NM_002053	-0.43495804
NM_024523	-0.509391321
NM_000161	-0.532348417
NM_001486	-0.610825972
AI559539_RC	-0.409586857
NM_002061	-0.40973614
NM_004864	-0.591712657
NM_020634	-0.426475136
NM_181702	-0.448933384
NM_017655	0.408301616
NM_000165	-0.524113436
Contig40478_RC	-0.546218935
NM_020198	-0.520996249
NM_000168	0.4541127
NM_002064	-0.451530289
Contig5961_RC	0.409665568
NM_006572	-0.444720349
NM_053004	-0.494036933
AK001469	-0.622191854
NM_000825	0.597253079
NM_152742	0.434815132
NM_014879	0.403237101
NM_138445	0.44197646
Contig45082_RC	-0.650623592
NM_153832	0.427515109
NM_001505	0.53928016
NM_005282	-0.621855399
NM_005304	-0.501956451
NM_006056	0.408070071
NM_032554	0.452293079
NM_018653	0.401454851
NM_013372	-0.425974097
NM_012203	0.44825581
NM_133445	0.463409316
NM_025196	-0.729407107
NM_152407	-0.611236299
NM_031485	-0.525886202
NM_173849	0.542890999
NM_002093	-0.535776296
NM_000177	0.593724122
NM_000178	0.408936054
NM_015917	0.418219157
NM_004128	-0.573052477
NM_012086	0.447998874
NM_012341	-0.764061198
NM_005317	0.489069429
NM_018267	0.462429993
NM_002105	-0.662932661
NM_005324	-0.441745045
NM_021175	-0.40460494
NM_003947	-0.433637877
NM_021817	0.498441516
NM_178232	-0.514285312
NM_001523	-0.639759507
NM_012257	0.532456438
NM_018684	0.56477739
NM_053005	0.449968627
NM_015416	0.487725844
AB040968	0.496189888
NM_002111	0.457866443
NM_006037	-0.659478185
NM_139205	0.589403887

NM_005474	0.544076018
NM_016073	-0.416597973
NM_006831	-0.685240964
NM_014877	0.499305124
NM_138737	0.413481336
NM_139004	0.475817174
NM_006553	-0.468437288
NM_000601	-0.449963237
NM_016258	-0.409185275
NM_002729	-0.514050738
NM_001529	-0.56607162
NM_153236	0.411327234
Contig40128_RC	-0.54793646
NM_001530	-0.648588522
NM_181054	-0.686727897
NM_022462	0.644912629
AB014555	0.464086484
Contig37871_RC	0.448345413
NM_021958	-0.49856785
NM_145899	-0.457004817
NM_002129	0.440319669
AF131827	0.577594449
NM_000859	-0.450208324
NM_002130	-0.62282955
NM_006353	-0.548075506
NM_002133	-0.718727192
NM_004499	-0.56120962
NM_031266	-0.609194651
Contig36638_RC	0.491711457
NM_004966	-0.688207372
NM_019597	-0.41576886
NM_005826	-0.403277506
NM_004272	-0.676654407
NM_015438	0.438146884
NM_000522	0.468581423
NM_153631	0.519531084
NM_004503	0.406464123
NM_006897	0.443949202
NM_000194	-0.581611724
NM_000861	-0.450850988
Contig53965_RC	-0.520429139
Contig55193_RC	-0.624406974
AJ251708	0.463086491
NM_014473	-0.648387378
NM_000196	0.439336419
NM_014234	0.46786591
NM_016299	-0.415462834
NM_005347	-0.471631317
NM_004134	-0.486500503
NM_016387	-0.408177843
NM_016391	-0.752133895
NM_016399	-0.526883669
NM_014173	0.4577436
NM_002156	-0.479381752
NM_002157	-0.439272779
NM_006644	-0.696586095
NM_013300	-0.510627652
AF086402	-0.585636482
NM_013285	-0.606626933
AJ276240	0.452524235
AJ227898	-0.525615882
NM_152553	-0.415048936
NM_153341	-0.541834801
NM_170705	0.429000099
M96843	-0.449562011

Contig47495_RC	-0.582207737
NM_015384	-0.416179809
NM_004907	-0.615234747
NM_003897	-0.665216458
NM_016545	-0.508919573
NM_005531	-0.717841575
NM_001551	0.458814921
NM_000597	0.478612851
L27560	0.618742436
NM_016291	0.644732296
NM_000572	-0.496322731
NM_000882	-0.547306019
NM_002188	-0.625989333
NM_000585	-0.431968589
NM_172217	0.629974735
NM_003855	-0.542090148
NM_000576	-0.635282487
NM_000877	-0.513022672
NM_000418	-0.617285508
NM_000600	-0.816483765
NM_000584	-0.604047649
NM_130759	0.442216878
NM_016162	0.708234653
NM_002193	-0.470969233
NM_005542	-0.586921519
NM_002198	-0.535592841
NM_002201	-0.676913124
NM_005545	0.459911985
NM_002205	-0.539779194
NM_000887	-0.440116044
NM_004867	0.401197493
NM_030926	0.523739624
NM_006469	-0.401951285
Contig55048_RC	0.465577964
NM_004653	0.542556737
NM_033626	0.471581818
NM_016604	0.440665388
AB002344	-0.528231874
NM_020655	0.508909544
NM_006303	-0.607732933
NM_002229	-0.657514554
NM_030929	0.508155603
AL117562	-0.506965394
AB067508	-0.439071423
NM_172160	0.42144849
NM_004979	0.408742571
NM_170736	-0.444168442
NM_002243	-0.584085603
NM_000891	-0.507445219
NM_003740	-0.542735157
NM_014407	0.515357459
Contig41578_RC	0.426204262
NM_031954	-0.445538556
NM_138444	-0.586718127
NM_002253	-0.423680628
NM_006558	-0.571300121
D26488	-0.644493849
NM_014878	-0.569775003
NM_014777	-0.644770458
NM_014661	0.575589617
NM_014773	0.422957102
NM_014738	0.536384631
D86985	-0.50055341
NM_014734	-0.492423906
D87470	0.521697376

NM_015324	-0.467947563
AB007915	-0.533465875
NM_024547	0.420981482
NM_014870	0.548299581
NM_015176	-0.526963198
AB007962	-0.596625496
AB011115	0.545344297
NM_014662	0.435494083
NM_015179	-0.648346249
NM_014719	0.546677096
AB018345	-0.510856436
NM_014924	0.471309267
NM_014963	-0.690312643
NM_014929	-0.523808408
NM_025164	-0.464269281
NM_014925	0.48908755
AB028949	-0.472372153
NM_014909	0.428778803
NM_015281	0.425310073
NM_015079	0.518083049
AB029030	0.418285606
AB032996	-0.432740817
AL137567	0.601882777
NM_017963	-0.427479229
NM_025081	0.43877554
AB037815	0.450617665
NM_020817	0.44871434
NM_017780	-0.511244942
NM_020832	0.417288362
AB040883	-0.50723923
Contig57398_RC	-0.593350192
Contig52095_RC	-0.433095156
AB040938	0.528517865
Contig49270_RC	-0.679584131
AB046843	0.430165481
AB051458	0.546400129
AB051470	0.570745507
Contig48436_RC	-0.509962663
Contig43542_RC	-0.413698961
AB051511	-0.507245743
NM_024935	0.405570606
NM_017794	0.608747207
AB058729	-0.427309078
AB058765	0.49779326
NM_052913	-0.515251331
NM_032550	0.548567251
Contig2263_RC	-0.507504849
NM_032873	-0.557966155
Contig924_RC	-0.583734415
NM_007250	0.436763148
NM_007246	-0.428074274
NM_017415	0.4464178
NM_130446	-0.592742225
NM_022046	-0.415310495
NM_002266	-0.663961644
NM_032107	0.566840811
NM_015478	0.404944361
NM_032857	-0.544131637
NM_025144	-0.455712262
NM_005559	-0.534935738
NM_000227	0.431981986
NM_018407	-0.509213829
NM_002296	-0.447360503
NM_014793	-0.654677218
NM_005565	-0.515224521

NM_000527	-0.704968202
AK026321	0.411318303
NM_139284	0.420457765
NM_024119	0.431823773
NM_006893	0.515322802
NM_005779	-0.476054249
NM_002309	-0.473408353
NM_006864	-0.477839136
NM_016733	-0.504587806
NM_005569	-0.528961031
NM_018362	-0.565334891
NM_006033	-0.571903597
NM_021250	-0.434670406
NM_004862	-0.565118602
NM_019081	0.442619306
NM_030805	0.460356653
NM_032737	-0.544869934
NM_002315	0.586332906
NM_005475	-0.423112435
NM_138422	-0.528662348
AL050205	-0.510592557
NM_052879	-0.725235387
Contig63304	-0.635023103
BC013767	-0.60021415
Contig38772_RC	0.413561626
BC017488	0.595055694
NM_145254	-0.541714518
NM_182752	0.435088446
NM_139016	0.419942912
NM_152994	0.575411525
NM_138285	-0.695680936
NM_178833	-0.643004125
BC016683	-0.429915618
Contig1815_RC	0.415327066
NM_138478	0.509359676
NM_181710	0.401994779
NM_144697	0.797578963
NM_175853	0.405022169
NM_178835	0.440926969
NM_145282	0.414232292
AI339981_RC	-0.494332672
NM_138491	0.515874229
Contig58107_RC	0.549026004
Contig40903_RC	-0.633521358
NM_174918	-0.551168882
AL110170	-0.408764898
NM_145309	0.412338762
Contig55984_RC	-0.481748555
NM_181719	0.452576926
NM_182556	0.533923613
NM_174940	0.433316713
NM_175897	0.428267984
NM_175898	-0.475373118
Contig53406_RC	-0.647114643
Contig32072_RC	-0.432308678
RSE_00000601195	0.565334274
Contig63026	-0.624090819
AW673036_RC	-0.532722168
Contig15283_RC	0.522030958
NM_178172	0.493375533
NM_178538	0.536815142
NM_178564	0.428003568
Contig6498	-0.625286781
ENST00000295549	0.53097474
Contig4595	-0.715510889

Contig44964_RC	-0.581685434
NM_016175	0.435289364
Contig57359_RC	-0.515785028
NM_016210	0.510156078
Contig41883_RC	0.452605314
NM_016496	0.465568238
NM_016644	0.496075312
NM_016647	0.468041283
NM_020142	0.462221384
NM_020143	-0.550549323
NM_020163	0.410097148
NM_020313	-0.522449238
NM_030802	0.559161048
NM_032509	-0.690120624
NM_053040	-0.657321169
NM_145232	-0.44823665
NM_138771	-0.466713933
NM_138363	0.456266774
BC006136	-0.593114842
NM_138385	0.405627795
NM_138386	-0.450078888
NM_138779	-0.693150357
NM_138397	-0.401923122
NM_018361	-0.519383728
NM_145886	0.489732465
NM_052972	-0.586897668
NM_181726	-0.685823523
NM_002337	0.421577755
NM_025168	0.526336484
NM_018205	0.466299182
NM_018334	0.445493851
NM_032808	0.530036071
NM_138361	0.461868709
NM_014462	-0.509450576
NM_032881	-0.413598715
NM_021070	0.40587475
NM_000897	0.515061622
NM_016153	0.589509759
NM_017816	-0.60568833
NM_014573	-0.492007489
Contig46202	-0.439963201
NM_014628	-0.442640986
NM_015583	0.462712798
NM_005902	-0.506305734
NM_012323	-0.674021326
NM_002359	-0.441931825
NM_014599	0.432688573
NM_030801	0.590773777
NM_173515	-0.572756486
NM_031419	-0.702351322
NM_025146	-0.448692136
NM_145160	0.457439968
Contig40090_RC	-0.416245515
NM_145333	-0.463297667
NM_006116	0.54657725
NM_005204	-0.507998479
NM_004579	0.523841995
NM_018194	0.472442498
AK054569	-0.681384227
NM_005911	-0.766708975
NM_002382	-0.483065458
NM_182763	-0.570691616
NM_006739	0.492318649
NM_005916	0.594617394
Contig48371	0.530304093

NM_020231	-0.501184593
NM_152305	-0.638266323
NM_018463	0.543197146
NM_018466	-0.497243465
NM_005466	0.498240108
NM_024102	-0.429209025
NM_022566	-0.690111961
NM_138395	-0.825030872
NM_005371	-0.688765837
NM_005927	-0.662344774
NM_030780	-0.563862468
NM_012214	-0.441533618
NM_031492	0.509564277
NM_033212	0.62814987
NM_032889	-0.568003994
NM_032683	-0.419150089
NM_152288	0.49820224
NM_032711	-0.492253543
NM_032356	0.421863992
NM_032334	-0.419073125
NM_032338	-0.485757706
NM_032369	0.408241748
NM_032756	-0.573048461
NM_053047	-0.606643602
BC007381	0.405374626
NM_052943	0.510799612
NM_052880	0.719017806
NM_080655	-0.570585199
NM_152452	0.449869173
NM_152421	0.524943099
NM_052857	-0.531944593
NM_138417	-0.500130546
NM_182614	0.404504108
Contig49520_RC	0.428012182
NM_032331	-0.604014753
NM_152430	0.458615229
U78519	-0.677346759
Contig60301_RC	0.403612173
NM_152783	0.597524475
NM_032631	0.52555459
NM_152339	-0.513328322
NM_032701	0.556358442
NM_032299	-0.497718731
NM_024297	-0.597507502
NM_031298	-0.544462964
NM_014388	-0.551572695
NM_144638	0.448475144
NM_032285	0.531639343
NM_023947	0.400788826
NM_153374	-0.477256209
NM_152435	0.424238245
NM_182550	0.411775046
NM_152318	-0.662053305
NM_152755	0.63751532
NM_153361	0.456842781
NM_031304	-0.465602929
NM_152711	-0.456041967
NM_182552	0.430522206
NM_024111	-0.432527273
NM_152398	0.492870428
NM_152464	-0.490237015
Contig29982_RC	0.501067612
NM_032326	0.458703165
NM_033309	0.449721274
NM_052871	-0.517687079

NM_032314	0.43789998
NM_178450	-0.520731059
NM_178540	0.526405782
NM_024116	-0.648282773
NM_032740	0.467898825
NM_024092	0.415919428
NM_016500	-0.551062956
NM_173638	0.418430156
NM_012215	0.574721387
AL512725	-0.715514652
NM_021242	-0.527734871
NM_032390	-0.783272114
NM_022443	-0.438120248
NM_005938	0.559032831
NM_023009	-0.462449921
NM_024101	0.425015548
NM_033467	0.585631292
AK057179	0.560056855
NM_002422	-0.618413502
NM_022362	0.419086888
Contig48290_RC	-0.503447152
Contig57496_RC	-0.458809955
NM_145279	-0.402415657
NM_005942	0.404106132
NM_019556	-0.515975742
NM_023948	0.477534775
NM_005791	-0.582621942
NM_032111	-0.582683324
NM_031903	-0.538754551
NM_172177	-0.453005978
NM_016055	0.404460229
NM_019051	-0.504349288
NM_015969	-0.457197942
NM_016067	0.411634847
NM_016034	-0.451633133
NM_016640	-0.422472581
NM_021821	-0.41462164
NM_032476	0.565000311
NM_005098	-0.450344612
NM_172165	0.476085403
NM_012331	0.464321611
NM_020998	0.505289002
NM_002447	0.516546996
NM_002448	-0.485629401
NM_002449	-0.413419788
NM_014507	-0.428826108
NM_005946	-0.66367864
NM_005951	-0.537352106
NM_175622	-0.51273312
NM_176870	-0.513367896
NM_002450	-0.426813741
NM_005952	-0.533992802
NM_005953	-0.628297825
NM_004739	-0.468829834
AB033092	0.59150967
NM_005955	-0.41287734
NM_006636	-0.638765126
NM_152912	0.42142225
NM_015458	-0.460808797
NM_173710	0.546521573
NM_138299	0.504564558
NM_032220	0.449820308
NM_006454	0.616368178
NM_004997	-0.610697192
NM_002467	-0.736700746

NM_002468	-0.403896284
NM_004998	-0.523036353
Contig31192_RC	-0.404469546
NM_014664	-0.487245231
NM_015909	0.523207188
NM_024865	0.467887271
Contig6323_RC	0.452444039
NM_003826	-0.41459008
Contig33758_RC	-0.502686064
NM_032041	0.609922217
AK054570	0.596744922
NM_002486	-0.435788573
NM_007362	-0.422803764
NM_080678	-0.416922212
NM_016453	0.546244516
NM_005381	-0.423038219
NM_024831	-0.603849054
NM_030808	-0.458420983
NM_138704	-0.594743661
NM_032013	0.607955716
NM_003635	-0.529842849
NM_015277	0.426090781
NM_024608	0.581051549
NM_152720	0.544211383
NM_014397	-0.728596954
NM_080741	-0.646138925
NM_006162	0.487550076
NM_006164	-0.534017864
NM_005384	-0.418873251
NM_002502	-0.518325285
NM_004556	-0.545945542
NM_014847	-0.619469918
NM_024946	0.492050409
NM_030922	-0.469293955
NM_007184	0.430708201
NM_033120	-0.535603468
NM_006167	-0.634055483
NM_000269	-0.453647322
NM_015039	0.545036472
NM_006169	-0.675463353
NM_014062	-0.586832465
NM_006067	-0.447082041
NM_006170	-0.72051043
NM_006392	-0.670627741
NM_139235	-0.568903133
NM_017948	-0.559728296
NM_018983	-0.591898561
NM_018648	-0.435579845
NM_004741	-0.726044051
NM_015934	-0.713667805
NM_020962	0.455383442
NM_052946	0.495184087
AF039697	0.43430908
NM_000270	-0.69672123
NM_000271	-0.594963187
NM_024663	0.410390192
NM_003717	0.478297459
NM_002523	-0.430081333
NM_002135	-0.682183927
NM_173198	-0.775955503
NM_002524	-0.461286318
NM_030759	-0.606106442
NM_017544	-0.620636696
NM_013981	0.61406487
NM_004883	0.612312822

NM_013982	0.600911208
NM_018534	-0.43741029
NM_014366	-0.673802168
NM_173474	0.409439482
NM_002528	0.548608335
NM_021229	0.404388439
NM_005013	0.403035128
NM_006185	0.618077394
NM_005124	-0.427173574
NM_153684	-0.447408974
NM_016320	-0.415279385
NM_139131	-0.667151827
NM_014778	-0.671048385
NM_013248	-0.769339544
NM_002542	0.401344933
NM_178014	0.433143747
NM_014279	0.417145953
NM_182487	0.465621306
NM_020190	0.473991478
NM_006812	0.466520132
NM_014835	0.475968514
NM_017731	0.492144869
NM_020530	-0.712305997
NM_053001	0.517179161
NM_178129	-0.435344638
NM_020804	0.428702054
NM_022490	-0.45661415
NM_017906	-0.760562067
NM_024594	-0.461964208
Contig51888_RC	-0.554812738
NM_015368	-0.68568502
NM_018109	-0.461653538
NM_007262	0.53665158
NM_182790	-0.428364479
NM_005746	-0.622991761
NM_025245	0.411878714
AL137559	0.611331204
NM_020418	0.403465348
NM_019035	-0.456338951
NM_000439	-0.567556034
Contig51403_RC	-0.539477055
NM_005017	-0.559492447
D80007	-0.613635803
NM_145341	0.563217074
Contig49768_RC	-0.50576173
NM_022341	-0.520172204
NM_033023	-0.550537869
NM_006207	0.411049867
NM_020992	-0.602591672
NM_003687	-0.465906632
NM_013377	0.42799845
NM_021830	-0.734844022
NM_014935	0.437912992
NM_002616	1
NM_014303	-0.514217344
NM_012394	-0.524867339
NM_004566	0.574231888
NM_000289	0.41178146
NM_170753	0.43068367
NM_032592	0.560287785
NM_004427	-0.484028304
AL117477	0.527950496
AK026181	-0.594369541
NM_006099	0.45822643
NM_004569	-0.638051782

NM_178517	-0.721128909
NM_003557	-0.622652765
NM_012417	-0.462316285
Contig43433_RC	-0.479685958
NM_024874	0.619926177
NM_000299	-0.500106566
NM_005090	0.588357764
NM_000929	0.499906718
NM_002657	-0.504516582
NM_000930	0.429138442
NM_002658	-0.578389701
NM_182811	0.449071654
NM_002663	0.548641425
AF131859	-0.43655014
NM_024310	0.716359931
AB033035	-0.47689672
AB002354	-0.485009744
NM_006622	-0.411611193
NM_004073	-0.532739752
NM_002669	-0.401276502
NM_021105	-0.602208721
NM_020360	0.450911971
NM_015103	0.4239946
NM_021127	-0.424919963
NM_000303	-0.622570478
NM_033109	-0.618514933
NM_002688	0.438245479
NM_004574	0.505934188
NM_153703	0.503113923
NM_017542	-0.658683399
NM_002690	0.450268293
NM_017443	-0.429190388
NM_004875	-0.454877837
NM_004805	-0.729564887
NM_018119	-0.465009757
NM_172020	-0.531500959
NM_007171	0.440035856
NM_000940	0.434593244
NM_015029	-0.684663582
NM_025204	-0.441920841
NM_152301	-0.529559448
NM_025201	-0.439011241
NM_020230	-0.655915485
NM_006238	-0.498414525
NM_177435	-0.625558237
NM_002703	-0.718930254
NM_177444	0.604229074
Contig42058_RC	0.402622081
NM_006112	0.470211279
NM_002705	0.605836191
NM_002714	-0.550277468
NM_014330	-0.512399504
Contig340_RC	-0.538324633
NM_015568	0.533478656
NM_024607	-0.413848806
NM_002715	-0.418899393
NM_015062	-0.734695081
NM_138934	0.429544143
NM_013239	0.565862727
NM_012406	-0.471453514
NM_012094	0.44607724
NM_002727	-0.417558621
NM_033405	-0.408025792
AK055479	0.598142016
NM_002730	0.424269156

Contig55540_RC	-0.400452729
NM_012408	0.68727537
NM_018137	-0.4900421
Contig37200_RC	-0.467233584
NM_014138	0.499153574
NM_018509	-0.515298671
NM_018621	0.428015747
NM_018520	0.530841973
NM_021935	-0.52745682
AK021524	0.45133069
NM_004697	-0.756818477
NM_017456	-0.54347888
NM_004762	-0.575790374
NM_002790	-0.539634154
NM_152255	-0.415418122
NM_005789	-0.64801611
NM_031991	-0.561247452
NM_031990	-0.562864963
NM_002819	-0.564442826
NM_175847	-0.567395755
NM_003463	-0.552849455
NM_003479	-0.416236003
NM_002827	-0.560939882
NM_014369	0.522776254
NM_080422	-0.560029808
NM_130435	-0.440644937
NM_130843	0.491594001
NM_002852	-0.765286445
Contig46241_RC	-0.452879481
AK057669	-0.540390344
NM_025215	-0.655972012
NM_006505	-0.698904059
NM_007062	-0.421751451
NM_005049	-0.645257648
NM_002859	-0.446173597
NM_181701	-0.573659219
NM_024638	-0.742720599
NM_017817	-0.43995171
Contig54258_RC	-0.436761387
NM_020673	-0.500497075
Contig32637_RC	-0.466623665
NM_022456	-0.46801184
NM_012197	0.509702794
NM_004582	-0.42138043
NM_013412	0.420668394
NM_007081	0.43150992
Contig46590	0.472256144
NM_134423	0.498281288
NM_003610	-0.430488153
NM_017574	-0.402080254
NM_015577	-0.505693021
NM_021785	0.408900467
AK055564	-0.66811332
NM_003979	-0.704480677
Contig19194_RC	-0.49532801
NM_015592	0.484852669
NM_006105	0.576090754
NM_012294	-0.522971471
NM_000964	-0.685871086
NM_170774	-0.450870197
NM_002894	-0.454156285
NM_022370	0.448188327
NM_022768	-0.580984668
NM_032905	-0.561988961
NM_022830	-0.406236627

NM_002896	-0.479798191
NM_016090	-0.425507291
NM_005105	-0.42320968
NM_016839	-0.439929771
NM_005772	-0.433463615
NM_021975	-0.411391789
NM_006509	-0.625917119
NM_014012	0.411495899
NM_018339	-0.49972912
NM_017819	-0.54191214
NM_002928	-0.680754898
NM_002923	-0.486980578
Contig44867_RC	-0.445559565
NM_033345	0.415338106
NM_014715	0.466349381
NM_018372	-0.425433281
NM_004292	-0.545533441
NM_153005	-0.708906237
NM_018343	-0.417971267
NM_003804	-0.516274206
NM_003821	-0.547743281
NM_015444	-0.638279059
NM_006912	-0.590006765
NM_002933	0.405666305
NM_002936	-0.523560169
Contig684_RC	-0.459549259
NM_024787	-0.563550377
NM_024778	-0.754696025
NM_173647	-0.417000086
NM_007212	-0.472942193
NM_002938	-0.504927608
NM_005977	-0.485361766
NM_025065	-0.437119406
NM_144563	-0.540474547
NM_000988	0.405337547
NM_021029	-0.553020712
NM_000973	0.566104005
NM_001002	0.574644578
NM_006638	-0.665582303
NM_005617	0.480140511
NM_015169	-0.717229931
RSE_00000862609	0.475188021
NM_001754	-0.698257821
NM_014328	0.450413134
AB007857	0.502820769
NM_002964	-0.442074799
NM_020368	-0.5817933
NM_002975	0.462420379
NM_006998	0.44235665
NM_006746	-0.428476525
NM_006089	-0.403329473
NM_174934	0.47671422
NM_005138	-0.561008247
NM_002999	-0.650692576
NM_006649	-0.694171692
NM_006643	-0.517846278
NM_022044	-0.421263498
NM_031216	-0.532682116
NM_032970	-0.408094217
NM_007190	-0.50281818
NM_015490	0.498106357
NM_013336	-0.411036746
NM_003004	-0.536767694
NM_000450	-0.60144956
NM_003944	0.410433725

NM_018445	-0.522238851
NM_004186	-0.560801057
NM_017789	-0.511400737
NM_152699	-0.648540381
NM_012248	-0.751403384
NM_030666	-0.456469564
NM_002575	-0.475308484
NM_002640	-0.668038512
NM_000602	-0.563594559
NM_013376	-0.735676145
NM_014755	-0.463406754
NM_003011	-0.468272643
NM_015559	0.490460438
NM_012432	0.415817521
NM_004630	-0.423298791
NM_005066	-0.553684037
NM_006924	-0.428445196
NM_004593	-0.581413885
NM_003016	-0.411814288
NM_003017	-0.568165022
NM_006276	-0.57475094
NM_005627	-0.507364628
NM_015503	0.425154284
NM_031469	0.40176193
NM_020145	0.581803919
NM_020870	-0.455800915
Contig60194_RC	-0.544753259
NM_020859	0.470759177
NM_173216	0.542634024
NM_018414	0.507811557
NM_152996	-0.534731992
AB037810	-0.480639467
NM_012240	0.487001831
NM_031244	0.594283839
NM_012241	0.441890969
NM_005982	0.404435204
NM_016932	-0.533716538
NM_175875	0.433520497
NM_016532	0.447689248
NM_003037	-0.484549717
AL117421	0.401849117
NM_006527	-0.531452884
NM_000617	-0.421181361
NM_004207	-0.534130462
NM_006996	-0.436706858
Contig44425_RC	-0.47373191
NM_005415	-0.657600806
NM_006749	-0.637609879
NM_016609	0.470148382
NM_007105	-0.519578002
NM_014251	-0.478442763
NM_014252	-0.527814732
NM_021734	-0.573007576
AK057476	0.520416988
NM_024698	-0.615534844
AB067483	-0.601560866
AK025078	-0.460875471
Contig58471_RC	0.431273408
NM_006516	-0.506758445
NM_153449	-0.581254475
NM_006931	-0.686631212
NM_020062	0.463370531
NM_003039	-0.629465665
NM_021194	-0.506453611
NM_017964	-0.450719811

Contig25949_RC	-0.403979578
NM_005827	-0.651358609
NM_015945	0.411987846
NM_182838	0.555957026
NM_014854	0.508817637
NM_017515	-0.492602342
NM_001467	0.402868767
NM_018573	-0.413376992
NM_018976	-0.418611789
D31887	-0.63131376
NM_002394	-0.574140046
NM_017836	0.519185601
NM_017611	-0.554548085
NM_003615	-0.732054534
AL050021	-0.791016195
NM_003486	-0.70052804
NM_004785	0.424023745
NM_173653	0.419773403
NM_016354	-0.416188961
NM_003072	-0.420773938
NM_139067	0.524116856
NM_015110	-0.474839185
NM_005631	0.468517006
NM_022739	-0.596464023
NM_173354	-0.613780536
NM_018664	-0.700513871
NM_024700	-0.413114488
NM_003498	-0.514307323
NM_003091	-0.52084464
NM_006938	-0.540598465
NM_003094	-0.447219679
NM_003095	-0.408113069
NM_003097	0.446267936
NM_005678	0.430539614
NM_013323	-0.544426148
NM_003101	-0.449295067
NM_003745	-0.533051419
NM_004232	-0.439083063
BC001980	-0.545552181
NM_000636	-0.611011864
NM_006942	0.495690452
NM_022454	-0.455082229
NM_031439	-0.635516535
NM_003971	-0.507054828
NM_024063	-0.532378522
NM_018418	0.457996387
NM_020239	-0.446256511
NM_021972	-0.643775825
NM_152594	-0.65509102
AF041037	-0.410554981
NM_030964	-0.540618584
NM_007173	-0.450851276
NM_003900	-0.401274451
NM_003131	-0.469746388
NM_003135	-0.463891004
NM_021203	-0.645763546
Contig14796_RC	-0.450421124
NM_016305	-0.519850016
NM_004600	-0.412728279
NM_080862	0.408654084
NM_017857	0.588952965
NM_024937	-0.443101051
NM_013437	-0.443947715
NM_003473	-0.442510391
NM_139164	-0.400639019

Contig57662_RC	-0.434538469
AB037721	0.613900439
NM_139276	-0.488492397
NM_003150	-0.587439813
NM_004760	-0.637740789
NM_006374	0.468234234
NM_004099	-0.422630925
NM_177424	-0.416523918
NM_001055	0.412588179
NM_003166	0.485380961
NM_177552	0.482909098
NM_019601	0.571261221
NM_004711	0.577311316
NM_006474	-0.558092238
NM_052864	-0.638251503
Contig16654_RC	-0.413643696
NM_006354	0.542360493
NM_139352	-0.432219001
Contig51821_RC	-0.450070051
NM_005641	0.424040813
NM_032505	-0.686207855
NM_133484	-0.401969989
NM_018009	0.457042849
NM_005646	0.406372284
NM_003191	-0.4192735
NM_015472	-0.651501601
NM_181313	0.427600097
NM_181314	0.417895118
NM_024682	0.545081222
NM_018421	-0.573619075
NM_025085	-0.65321103
NM_013254	-0.418193375
NM_032988	-0.534018696
NM_003198	-0.596520071
NM_003199	0.45016726
NM_031283	0.441148681
NM_003211	-0.588956051
NM_003213	-0.703340181
NM_170754	0.431268039
NM_013319	-0.596539515
NM_001120	0.472409078
NM_017746	-0.640727525
NM_022366	-0.433518459
NM_012143	-0.4708206
NM_003234	-0.463623213
NM_003236	-0.672728603
NM_003239	-0.416388957
AK002171	-0.463690122
NM_000361	-0.573510074
NM_003247	-0.430576685
NM_007112	0.534947252
NM_005782	-0.530485462
AK054894	-0.404182047
NM_005121	-0.40643676
NM_005655	-0.409289568
NM_032862	-0.431351853
NM_012460	-0.439116742
NM_003254	-0.40028845
NM_015508	-0.426983187
NM_006289	0.408647174
NM_003268	0.400882023
NM_003272	-0.457649777
NM_013390	-0.715811332
NM_032780	0.483323846
NM_016456	0.491971147

NM_181428	0.629772941
NM_002160	-0.552214722
NM_000594	-0.5893372
NM_006291	-0.480297463
NM_007115	-0.504384461
NM_147187	-0.546359451
Contig54206_RC	-0.474063408
NM_016639	-0.531352361
NM_001065	-0.617327884
NM_003809	0.498372068
NM_024309	-0.433777183
NM_014494	0.438044159
NM_016272	0.428250494
NM_019009	-0.468150939
NM_006114	-0.59000563
NM_003286	-0.435622624
NM_005426	-0.548217156
NM_018475	-0.536846892
NM_006670	-0.40340949
NM_003288	-0.713768734
Z36778	-0.621093613
NM_003290	-0.556691409
NM_014317	-0.465414251
NM_013315	0.450552671
NM_007064	-0.555253261
NM_145725	-0.409112745
NM_003300	-0.539921862
NM_004295	-0.510519763
NM_012288	-0.436570582
NM_018643	-0.517705111
NM_145273	0.490851909
NM_018415	0.552365476
NM_025195	-0.709719285
NM_021158	-0.675831239
NM_005082	-0.422866524
NM_021616	0.455511439
NM_033549	0.425991754
NM_025188	0.452062468
NM_033093	0.447463977
NM_178125	0.524044272
NM_016157	0.601792588
NM_003303	0.52357511
NM_015638	0.417661467
NM_017636	0.45653737
NM_018727	0.426590168
NM_021055	0.463092964
NM_005725	0.423270319
NM_005706	-0.477021361
NM_004623	-0.66263989
NM_012263	0.576249699
NM_014640	-0.409343901
NM_133378	0.54714927
Contig2099_RC	-0.432249721
NM_032704	-0.466938204
Contig57226_RC	-0.4747845
NM_006086	-0.48375736
NM_016262	-0.491506615
NM_016437	0.561025006
Contig46324_RC	0.474467677
NM_006545	0.51336594
Contig35700_RC	-0.525003126
NM_006472	0.491223091
NM_003330	-0.630307131
U17626	-0.567105251
NM_014014	0.50926719

NM_018003	0.636885067
NM_003115	-0.502401139
NM_016525	-0.43217959
NM_003335	0.40627262
NM_003340	-0.519810238
NM_016336	-0.483822039
NM_016405	-0.445506481
NM_003347	0.421859793
NM_003348	-0.435406227
NM_017582	-0.591477456
NM_006048	0.453002892
NM_014517	-0.466693162
NM_053067	-0.593924878
NM_013438	-0.594043185
AF289595	-0.422450163
NM_003359	-0.504677146
NM_025217	-0.43463092
Contig47339_RC	-0.652220584
NM_012474	-0.744003488
NM_005148	0.407412426
NM_080911	0.448694741
NM_181597	-0.65091339
NM_000374	0.411026541
NM_031941	0.470683932
NM_182488	-0.450741303
NM_017414	0.472655496
Contig42760_RC	-0.472388635
NM_025090	-0.476890342
NM_032557	-0.483687158
AK001647	0.420163696
Contig62923_RC	-0.428666971
NM_014871	0.500813686
NM_003574	-0.58191463
NM_003370	-0.528202192
NM_000376	-0.634665983
NM_005429	-0.459232493
NM_030938	-0.572969114
NM_004665	-0.40192426
Contig46224_RC	-0.502647686
NM_080432	-0.411749898
NM_015289	0.450503961
NM_004184	-0.446687547
NM_012477	0.543732351
NM_175064	0.587075493
Contig39451	0.436368258
NM_005112	-0.47504225
NM_018256	-0.695111653
NM_181340	-0.527482742
NM_025160	-0.435077225
NM_139281	-0.582714794
NM_033662	-0.546820716
NM_033661	-0.62158993
NM_003880	0.486565064
NM_003391	-0.51428029
NM_006522	0.453703135
NM_004906	-0.557447434
NM_152857	-0.621267764
NM_130788	0.437577054
NM_005080	-0.671034014
NM_020750	-0.710616363
NM_012255	-0.444064396
NM_022167	0.438995959
NM_012479	0.612463402
NM_003405	-0.480623308
NM_152444	0.443029301

NM_016653	-0.517762434
NM_014838	-0.658517607
NM_152735	-0.554596966
NM_024625	-0.452429038
NM_017665	-0.593555186
NM_017742	-0.462526634
NM_024630	0.509339131
NM_024721	0.49807034
NM_003407	-0.404480903
NM_014733	-0.477777778
NM_032850	0.478690305
NM_024071	0.49092633
NM_178451	0.436742131
NM_138462	-0.471437783
U09410	-0.434923398
NM_006006	0.759698961
NM_007145	-0.687335647
NM_003451	0.426661254
NM_152736	0.501854407
NM_007152	-0.437606047
NM_003457	-0.493237887
NM_013398	0.469444512
NM_016444	0.419865129
Contig48954_RC	0.514249111
NM_005674	-0.69408861
AK000435	0.401328622
NM_005774	0.472128878
NM_003904	-0.601028508
NM_019591	-0.498420324
NM_005096	0.596204626
NM_005741	-0.588322966
NM_003414	-0.421393819
NM_012482	-0.408364387
Contig28228	0.547373652
NM_019110	0.412295282
NM_018683	-0.44001161
NM_014487	-0.469060062
NM_022482	-0.572964503
NM_030580	0.420316581
NM_005649	-0.634509258
NM_058230	-0.477124802
NM_018660	0.44230271
NM_003422	0.426517015
NM_030634	-0.419039423
NM_014797	-0.443844243
Contig43253_RC	-0.526510843
NM_145312	-0.54036081
NM_145291	-0.401259283
NM_015461	0.445132736
NM_032423	0.419482388
NM_152303	0.406077203
NM_024341	-0.656254162
NM_017656	-0.552810452
NM_152412	0.421075273
NM_152360	0.449854838
NM_024327	-0.600149898
NM_173548	-0.675744659
NM_015871	-0.468622283
Contig2728_RC	-0.542610093
NM_178167	-0.548639589
NM_032689	0.400104897
AB075832	0.53387601
NM_003427	0.483912966
NM_003428	-0.450608123
NM_170783	-0.642547775

NM_147128	-0.502567747
X98260	-0.573696154
Contig26988_RC	0.661181818
Contig26760	0.610995176
Contig12369_RC	0.606443235
Contig32577_RC	0.604849298
AK000802	0.597059643
Contig28286_RC	0.59440912
Contig29226_RC	0.587053109
Contig23198_RC	0.586063799
Contig36902_RC	0.583571924
Contig39408_RC	0.567382824
Contig34395_RC	0.551402552
Contig37895_RC	0.549341532
Contig34872_RC	0.545325387
Contig48430_RC	0.540798777
AF052101	0.532650191
Contig34701_RC	0.524253948
Contig37142_RC	0.521394385
Contig27084_RC	0.516708125
Contig16786_RC	0.513778912
Contig35897_RC	0.507316342
Contig22534_RC	0.507314591
AL049337	0.501576194
AI401061_RC	0.496110515
Contig45970_RC	0.495832759
Contig45303_RC	0.492052158
Contig21679_RC	0.491458426
Contig33003_RC	0.486242254
AF052138	0.48606201
Contig29513_RC	0.483953802
Contig38647_RC	0.480061606
Contig53177_RC	0.476187008
Contig44987_RC	0.474930124
Contig12814_RC	0.474903433
Contig50731_RC	0.471468508
Contig29380_RC	0.468679316
Contig56689_RC	0.468201977
Contig64477	0.464723303
Contig30955_RC	0.463759031
Contig23408_RC	0.46138933
Contig45914_RC	0.458929405
Contig24308_RC	0.457014891
Contig37761	0.453578459
Contig38914_RC	0.453130742
Contig13300_RC	0.452865958
Contig53342_RC	0.452793682
AL049279	0.451609253
AL050145	0.451144025
Contig19384_RC	0.448689174
Contig30213_RC	0.441700593
Contig10844_RC	0.437016684
Contig24152_RC	0.432098211
AF035318	0.43208524
Contig29022_RC	0.431382556
Contig25075_RC	0.431318035
Contig26077_RC	0.431018298
Contig42038_RC	0.431015509
Contig21421_RC	0.425092744
Contig36931_RC	0.424803681
Contig32810	0.422149675
Contig48722_RC	0.420529876
AF052119	0.420226231
Contig58121_RC	0.418652808
Contig15381_RC	0.417455023

Contig32798_RC	0.41730218
Contig34112_RC	0.41683239
Contig33574_RC	0.414601936
Contig48076_RC	0.41432023
Contig15693_RC	0.413342263
Contig52463_RC	0.413104891
Contig297_RC	0.406534915
Contig31596_RC	0.402322904
AL137723	0.401470524
Contig47247_RC	0.400971629
Contig24259_RC	-0.409628464
AL157488	-0.415873279
Contig50584_RC	-0.418463338
Contig51292_RC	-0.418919002
Contig18246_RC	-0.420962148
Contig55785_RC	-0.426244011
AK021516	-0.43067411
Contig16192_RC	-0.441903058
Contig31872_RC	-0.443803132
Contig21370_RC	-0.44466935
AL359055	-0.450262694
Contig45298_RC	-0.458078659
Contig47381_RC	-0.46834281
Contig59080	-0.475883335
Contig51486_RC	-0.482995409
Contig688_RC	-0.483485346
Contig37042_RC	-0.506225915
Contig46563_RC	-0.515788169
Contig35400_RC	-0.522519193
Contig45549_RC	-0.529786997
Contig66868_RC	-0.532640926
Contig111_RC	-0.539482129
Contig36419_RC	-0.540799008
Contig51764_RC	-0.545862923
Contig51066_RC	-0.551908027
Contig21997_RC	-0.55675907
Contig47879_RC	-0.577979582
Contig43853_RC	-0.585586709
Contig13480_RC	-0.592184549
Contig23085_RC	-0.59656151
Contig48806_RC	-0.604500591
Contig26835_RC	-0.625611852
Contig58279_RC	-0.673765166
Contig40785_RC	-0.682206604
Contig47512_RC	-0.70157899
