# Supplementary material for: Diurnal variation of the human adipose transcriptome and the link to metabolic disease
Source: BMC Med Genomics. 2009 Feb 9;2:7. doi: 10.1186/1755-8794-2-7 (PMC2647943; doi:10.1186/1755-8794-2-7)
Supplement: Additional file 9 — Transcripts that were found in previous studies to be significantly regulated by various growth factors in mammalian cell lines. [file 1755-8794-2-7-S9.pdf]

Symbol	UGCluster	Name	LLID	UGRepAcc
Genes upregulated with growth factors				
DDAH1	Hs.379858	Dimethylarginine dimethylaminohydrolase 1	23576	NM_012137
RQCD1	Hs.148767	RCD1 required for cell differentiation1 homolog (S. pombe)	9125	BM925206
CSTF2	Hs.132370	"Cleavage stimulation factor, 3' pre-RNA, subunit 2, 64kDa"	1478	AK095684
EEF1E1	Hs.631818	Eukaryotic translation elongation factor 1 epsilon 1	9521	BC005291
PSMA4	Hs.251531	"Proteasome (prosome, macropain) subunit, alpha type, 4"	5685	BC030529
PSMA3	Hs.558799	"Proteasome (prosome, macropain) subunit, alpha type, 3"	5684	BM918616
PSMB1	Hs.352768	"Proteasome (prosome, macropain) subunit, beta type, 1"	5689	AB209078
ODC1	Hs.467701	Ornithine decarboxylase 1	4953	CR614398
EIF5A	Hs.534314	Eukaryotic translation initiation factor 5A	1984	CR622789
UCHL3	Hs.162241	Ubiquitin carboxyl-terminal esterase L3 (ubiquitin thiolesterase)	7347	BF217744
FAM98A	Hs.468140	"Family with sequence similarity 98, member A"	25940	AK096187
PFDN2	Hs.492516	Prefoldin subunit 2	5202	BF203500
NARG1	Hs.555985	NMDA receptor regulated 1	80155	NM_057175
EXOSC3	Hs.591076	Exosome component 3	51010	NM_016042
UBE2V2	Hs.491695	Ubiquitin-conjugating enzyme E2 variant 2	7336	AK094617
DPH2	Hs.632398	DPH2 homolog (S. cerevisiae)	1802	NM_001384
SLC25A32	Hs.654812	"Solute carrier family 25, member 32"	81034	NM_030780
MRPS23	Hs.5836	Mitochondrial ribosomal protein S23	51649	BE782112
PSMC4	Hs.211594	"Proteasome (prosome, macropain) 26S subunit, ATPase, 4"	5704	CR611800
KBTBD6	Hs.534040	Kelch repeat and BTB (POZ) domain containing 6	89890	NM_152903
SUB1	Hs.229641	SUB1 homolog (S. cerevisiae)	10923	BX537584
NIP7	Hs.585728	Nuclear import 7 homolog (S. cerevisiae)	51388	NM_016101
MRPL50	Hs.288224	Mitochondrial ribosomal protein L50	54534	BE893534
TPI1	Hs.524219	Triosephosphate isomerase 1	7167	BM913099
CCNA2	Hs.58974	Cyclin A2	890	CR604810
GART	Hs.473648	"Phosphoribosylglycinamide formyltransferase, phosphoribosylglycinamide synthetase, phosphoribosylaminoimidazole synthetase"	2618	BC068438
MYC	Hs.202453	V-myc myelocytomatosis viral oncogene homolog (avian)	4609	NM_002467
RANBP1	Hs.24763	RAN binding protein 1	5902	AK094410
CDC45L	Hs.474217	CDC45 cell division cycle 45-like (S. cerevisiae)	8318	NM_003504
RAD54L	Hs.653212	RAD54-like (S. cerevisiae)	8438	NM_003579
ATAD3A	Hs.655253	"ATPase family, AAA domain containing 3A"	55210	AK092833
LDHA	Hs.2795	Lactate dehydrogenase A	3939	BM457440
IFRD2	Hs.315177	Interferon-related developmental regulator 2	7866	Y12395
EXOSC8	Hs.294041	Exosome component 8	11340	AK096810
DLEU2	Hs.547964	"Deleted in lymphocytic leukemia, 2"	8847	AF264787
TAF1A	Hs.153088	"TATA box binding protein (TBP)-associated factor, RNA polymerase I, A, 48kDa"	9015	NM_005681
ATP1B1	Hs.291196	"ATPase, Na+/K+ transporting, beta 1 polypeptide"	481	NM_001677
E2F7	Hs.416375	E2F transcription factor 7	144455	AK096316
UCK2	Hs.458360	Uridine-cytidine kinase 2	7371	BX640859
CTPS	Hs.473087	CTP synthase	1503	BC009408

NOLC1 Hs.523238 Nucleolar and coiled-body phosphoprotein 1 9221
D21262
MCM7 Hs.438720 Minichromosome maintenance complex component 7 4176
NM_182776
CDCA4 Hs.34045 Cell division cycle associated 4 55038 BG354577
MCM10 Hs.198363 Minichromosome maintenance complex component 10 55388
AL136840
LETM1 Hs.120165 Leucine zipper-EF-hand containing transmembrane protein 1
3954 BX537709
TRIP13 Hs.436187 Thyroid hormone receptor interactor 13 9319 NM_004237
HNRPAB Hs.248746 Heterogeneous nuclear ribonucleoprotein A/B 3182
AK123488
NUP155 Hs.547696 Nucleoporin 155kDa 9631 BC039257
CSE1L Hs.90073 CSE1 chromosome segregation 1-like (yeast) 1434
NM_001316
WDR62 Hs.116244 WD repeat domain 62 284403 BC058939
SHCBP1 Hs.123253 SHC SH2-domain binding protein 1 79801 BC030699
DTYMK Hs.471873 Deoxythymidylate kinase (thymidylate kinase) 1841
AF258562
DKC1 Hs.4747 "Dyskeratosis congenita 1, dyskerin" 1736 BC009928
ZWINT Hs.591363 ZW10 interactor 11130 CR624092
ZWILCH Hs.21331 "Zwilch, kinetochore associated, homolog (Drosophila)"
55055 BX640701
TNFRSF1B Hs.256278 "Tumor necrosis factor receptor superfamily, member
1B" 7133 BC052977
FABP5 Hs.408061 Fatty acid binding protein 5 (psoriasis-associated) 2171
BM563703
PNPT1 Hs.388733 Polyribonucleotide nucleotidyltransferase 1 87178
BC053660
TOMM40 Hs.655909 Translocase of outer mitochondrial membrane 40 homolog
(yeast) 10452 BC047528
NNMT Hs.503911 Nicotinamide N-methyltransferase 4837 AK097984
HLA-DMB Hs.654428 "Major histocompatibility complex, class II, DM beta" 3109
AB209577
KIAA1199 Hs.459088 KIAA1199 57214 AB103330

Genes downregulated with growth factors
CTDSP2 Hs.524530 "CTD (carboxy-terminal domain, RNA polymerase II,
polypeptide A) small phosphatase 2" 10106 NM_005730
CHES1 Hs.434286 Forkhead box N3 1112 AK055175
CCNG2 Hs.13291 Cyclin G2 901 BC032518
APLP2 Hs.370247 Amyloid beta (A4) precursor-like protein 2 334
BX647107
SEPP1 Hs.275775 "Selenoprotein P, plasma, 1" 6414 BC030009
PPP2R5C Hs.368264 "Protein phosphatase 2, regulatory subunit B', gamma
isoform" 5527 NM_002719
PINK1 Hs.389171 PTEN induced putative kinase 1 65018 AB053323
LRRC1 Hs.646997 Leucine rich repeat containing 1 55227 AU119761
MST1 Hs.349110 Macrophage stimulating 1 (hepatocyte growth factor-like)
4485 BC044862
FAM53B Hs.129195 "Family with sequence similarity 53, member B" 9679
NM_014661
PCK1 Hs.1872 Phosphoenolpyruvate carboxykinase 1 (soluble) 5105 BX648510
TRAK1 Hs.535711 "Trafficking protein, kinesin binding 1" 22906
NM_001042646
ZFYVE1 Hs.335106 "Zinc finger, FYVE domain containing 1" 53349 BC053520
BCL6 Hs.478588 B-cell CLL/lymphoma 6 (zinc finger protein 51) 604
BX649185
GBP2 Hs.386567 "Guanylate binding protein 2, interferon-inducible" 2634
NM_004120
MXD4 Hs.655020 MAX dimerization protein 4 10608 AK024501
HBP1 Hs.162032 HMG-box transcription factor 1 26959 NM_012257
MLLT7 Hs.584654 Forkhead box O4 4303 NM_005938
SSBP2 Hs.102735 Single-stranded DNA binding protein 2 23635 NM_012446

HIST2H2BE	Hs.2178	"Histone cluster 2, H2be"	8349	BC069193
BMF	Hs.591104	Bcl2 modifying factor	90427	NM_001003940
GRAMD1C	Hs.24583	GRAM domain containing 1C	54762	AL133661
METTL7A	Hs.655369	Methyltransferase like 7A	25840	NM_014033
FBXO15	Hs.664011	F-box protein 15	201456	AK093252
FAM63A	Hs.3346	"Family with sequence similarity 63, member A"	55793	AB037811
FANK1	Hs.352591	Fibronectin type III and ankyrin repeat domains 1	92565	CR627249
CCT6B	Hs.73072	"Chaperonin containing TCP1, subunit 6B (zeta 2)"	10693	CR933688
ING4	Hs.524210	"Inhibitor of growth family, member 4"	51147	NM_016162
YPEL2	Hs.463613	Yippee-like 2 (Drosophila)	388403	NM_001005404
PCDHB14	Hs.658497	Protocadherin beta 14	56122	BC050417
KLHL24	Hs.407709	Kelch-like 24 (Drosophila)	54800	NM_017644
PNRC1	Hs.75969	Proline-rich nuclear receptor coactivator 1	10957	NM_006813
TMC4	Hs.355126	Transmembrane channel-like 4	147798	BC025323
HHAT	Hs.58650	Hedgehog acyltransferase	55733	BC051191
CHGB	Hs.516874	Chromogranin B (secretogranin 1)	1114	BC000375
ARNT2	Hs.459070	Aryl-hydrocarbon receptor nuclear translocator 2	9915	NM_014862
ACSS1	Hs.529353	Acyl-CoA synthetase short-chain family member 1	84532	AK125058
GSTA2	Hs.94107	Glutathione S-transferase A2	2939	BI762502
